# Microbiome-Derived Metabolic Signaling in the Gut–Skin Axis: Implications for Nutricosmetics and Healthy Skin Aging

**DOI:** 10.3390/nu18183032

**Published:** 2026-09-16

**Authors:** Jihye Lee, Hae-Jin Park, Eun-Mi Ahn, Jung Min Cho, A. M. Abd El-Aty, Jaehoon Bae

**Affiliations:** 1 Hybrid Cosmetics Research Institute, Industry-University Cooperation Foundation, Daegu Haany University, Gyeongsan-si 38610, Republic of Korea; jhlee86@dhu.ac.kr; 2 Department of Foodcare YAKSUN, Daegu Haany University, Gyeongsan-si 38610, Republic of Korea; hjpark@dhu.ac.kr; 3 Functional Food Research Institute, Industry-University Cooperation Foundation, Daegu Haany University, Gyeongsan-si 38610, Republic of Korea; ahnem@dhu.ac.kr (E.-M.A.); jungmincho@dhu.ac.kr (J.M.C.); 4 Department of Food Science and Biotechnology, Daegu Haany University, Gyeongsan-si 38610, Republic of Korea; 5 Department of Pharmacology, Faculty of Veterinary Medicine, Cairo University, Giza 12211, Egypt; 6 Department of Medical Pharmacology, Faculty of Medicine, Ataturk University, Erzurum 25240, Türkiye; 7 Department of Food Industry, Daegu Haany University, Gyeongsan-si 38610, Republic of Korea

**Keywords:** gut–skin axis, microbial metabolites, nutricosmetics, skin aging, host–microbiome cometabolism, microbiome-targeted interventions, precision nutrition

## Abstract

The gut–skin axis represents a bidirectional communication network linking the gut microbiota, microbial metabolism, immune regulation, and skin physiology. Natural bioactives derived from dietary plants, mushrooms, fermented foods, marine resources, and functional ingredients may influence skin health through both direct nutritional effects and host–microbiome metabolic interactions. This review provides a microbiome-centered, systems-level perspective on the relationships among dietary bioactives, gut microbial biotransformation, microbiome-derived metabolites, and skin aging-associated physiology. Particular attention is given to polyphenols, polysaccharides, carotenoids, peptides, fatty acids, probiotics, postbiotics, and fermented bioactive compounds as modulators of microbial composition and metabolic activity. Microbial metabolites, including short-chain fatty acids (SCFAs), indole derivatives, urolithins, phenolic metabolites, bile acid derivatives, and lipid-associated metabolites, are discussed as potential mediators linking diet and microbial metabolism with skin physiology. Their relevance to oxidative stress, inflammation, mitochondrial function, epidermal barrier homeostasis, extracellular matrix remodeling, and cellular senescence is critically evaluated according to the available level of evidence. Human intervention studies provide preliminary clinical support for selected probiotic and oral nutricosmetic interventions; however, direct evidence establishing microbiome-mediated effects on skin outcomes remains limited. Interindividual differences in microbial metabolic capacity may further contribute to heterogeneous responses to dietary interventions. Overall, this evidence-centered appraisal reveals a substantial gap between mechanistic plausibility and demonstrated human causality, emphasizing the need for studies that directly test microbiome mediation using integrated microbiome, metabolite, and skin-specific outcome measurements.

## 1. Introduction

Skin health is increasingly recognized as an integral component of overall physiological well-being and healthy aging, extending beyond a cosmetic appearance to include epidermal barrier integrity, immune balance, tissue resilience, and systemic homeostasis [1,2,3]. Environmental stressors, dietary imbalance, psychological stress, pollution, ultraviolet (UV) radiation, and aging-related physiological changes can collectively contribute to epidermal dysfunction, chronic inflammation, and accelerated skin aging [1,3]. Consequently, nutritional and microbiome-targeted strategies that support skin homeostasis and healthy aging have attracted increasing amounts of scientific and industrial attention [4].

Recent advances in microbiome science have highlighted the gut–skin axis as a complex bidirectional communication network that links the gut microbiota, microbial metabolism, immune signaling, epithelial regulation, and skin physiology [5,6,7]. Alterations in gut microbial composition and metabolic activity may influence systemic inflammatory tone, oxidative stress, epidermal barrier function, and age-related cutaneous changes [6,7]. Conversely, skin disorders, environmental stress, and neuroendocrine responses may also affect systemic and microbial homeostasis, underscoring the dynamic nature of gut–skin communication [5,6].

Natural bioactive compounds derived from dietary plants, mushrooms, fermented foods, marine resources, and functional ingredients have attracted considerable interest as modulators of the gut microbiota and skin-related physiological pathways [8,9,10]. Compounds such as polyphenols, polysaccharides, carotenoids, peptides, fatty acids, probiotics, and postbiotics may influence the gut microbial composition and metabolic activity, thereby promoting the generation of bioactive microbial metabolites involved in gut–skin communication [8,9,10]. Importantly, the biological effects of many dietary bioactives may depend not only on their parent structures but also on host–microbiome cometabolism and individual microbial metabolic capacity [8,11,12]. Among gut-derived metabolites, short-chain fatty acids (SCFAs), indole derivatives, urolithins, phenolic metabolites, bile acid derivatives, and lipid-associated metabolites are being increasingly recognized as key mediators of immune regulation, epithelial signaling, oxidative stress responses, mitochondrial homeostasis, and extracellular matrix remodeling [11,12,13]. These metabolite-driven signaling networks may contribute to skin resilience, barrier integrity, and aging-associated cutaneous homeostasis [11,12,13].

Despite the rapidly expanding literature on the gut–skin axis, an important unresolved issue is not simply which biological pathways have been proposed but how strongly each step of the proposed diet–microbiome–metabolite–skin pathway is supported by experimental and human evidence [5,6,7,11,12,13]. Existing mechanistic models increasingly connect dietary bioactives, microbial biotransformation, microbiome-derived metabolites, host signaling, and skin-related outcomes; however, the evidence supporting these individual links is highly uneven [11,12,13,14,15]. In particular, evidence that a dietary compound alters the microbial composition, that a microbial metabolite affects a relevant signaling pathway, or that oral intervention improves a skin endpoint does not, by itself, demonstrate that the observed skin effect is mediated by the gut microbiome. Establishing such a mediation requires evidence connecting microbial alteration or biotransformation, relevant metabolite exposure, and skin-specific outcomes within an appropriately designed experimental or clinical framework [15,16,17,18,19].

Accordingly, the present review does not consider the gut–skin axis, host–microbiome cometabolism, or metabolite-centered signaling to be intrinsically novel concepts. Instead, these concepts are used as an organizing framework to address a more restrictive question: which proposed diet–microbiome–metabolite–skin relationships are supported by mechanistic evidence, human associative evidence, intervention studies, or direct evidence of microbiome-mediated effects? Particular emphasis is placed on identifying discontinuities in the proposed causal chain and distinguishing biological plausibility from clinically demonstrated effects. From this perspective, a major translational gap in the current literature is the scarcity of human studies that simultaneously assess dietary exposure or intervention, microbial alteration or biotransformation, circulating microbiome-derived metabolites, and standardized skin-specific outcomes [16,17,18,19,20,21,22,23,24,25,26,27]. This evidence-centered appraisal is intended to clarify which components of the proposed gut–skin pathways are supported by current evidence, which remain inferential, and what evidence is required to move the field from association toward causality.

## 2. Literature Search Strategy

This article presents a narrative review supported by a structured, nonsystematic literature search aimed at providing an integrative and critical synthesis of mechanistic, clinical and translational evidence related to microbiome-derived metabolic signaling in the gut–skin axis. The relevant literature was identified through PubMed, Scopus, and Web of Science using combinations of terms such as “gut–skin axis,” “gut microbiota,” “gut microbiome,” “microbial metabolites,” “skin aging,” “nutricosmetics,” “natural bioactives,” “probiotics,” “postbiotics,” “short-chain fatty acids,” “indoles,” “tryptophan metabolites,” “urolithins,” “bile acids,” “oxidative stress,” “inflammation,” “mitochondrial function,” “skin barrier,” “precision nutrition,” “multiomics,” and “personalized nutrition.” No predefined lower publication-date limit was applied, and the literature search was updated through August 2026 to incorporate the most recent relevant evidence available at the time of manuscript revision.

Peer-reviewed original studies, including *in vitro* studies, animal models, observational human studies, and human interventional studies, were considered when relevant to the mechanistic, clinical, or translational scope of the review. Additional relevant systematic reviews, meta-analyses, narrative reviews, consensus statements, and methodological papers were considered to provide broader context and support the interpretation of established and emerging concepts. Priority was given to studies directly addressing relationships among dietary bioactives, gut microbial composition or metabolism, microbiome-derived metabolites, and skin-related physiological or clinical outcomes. Studies without clear relevance to gut–skin communication, microbial metabolic signaling, skin physiology, or the translational scope of nutricosmetic interventions were not prioritized for inclusion.

Because the objective of this narrative review was to provide a broad integrative synthesis rather than an exhaustive quantitative assessment of a predefined clinical question, no formal meta-analysis or risk-of-bias assessment was performed. The literature was synthesized according to major thematic domains, including natural bioactives, microbiome-derived metabolites, mechanistic pathways, human clinical evidence, and emerging precision nutricosmetic approaches. Greater interpretative weight was given to human interventional evidence when available, whereas observational, animal, and *in vitro* findings were interpreted according to their respective evidentiary limitations. The interpretation of the available evidence considered the study design, directness to skin-related outcomes, consistency of findings, and translational relevance, which is consistent with the narrative evidence-appraisal framework presented in Table 1.

## 3. The Gut–Skin Axis: Biological Basis and Emerging Concepts

### 3.1. Overview of the Gut–Skin Axis

The gut–skin axis refers to a complex bidirectional communication network that links the gastrointestinal tract, the gut microbiota, immune regulation, metabolic signaling, and skin physiology [5,6,7]. Increasing evidence suggests that intestinal homeostasis and gut microbial composition may be associated with epidermal barrier integrity, inflammatory balance, oxidative stress responses, and aging-associated skin physiology [5,6,7,11]. Conversely, skin disorders, environmental stressors, and neuroendocrine responses may also alter gut microbial ecology through immune-mediated and hormonal pathways, highlighting the dynamic and interconnected nature of gut–skin communication [5,6,7].

The human gastrointestinal tract harbors a highly diverse microbial ecosystem composed of bacteria, fungi, viruses, and archaea that collectively contribute to nutrient metabolism, immune maturation, epithelial homeostasis, and systemic metabolic regulation [4,11,69,70]. Dysbiosis, characterized by microbial imbalance and reduced microbial diversity, is associated with increased intestinal permeability, chronic low-grade inflammation, and altered microbial metabolite production [11,71,72]. These alterations are increasingly implicated in the pathogenesis of inflammatory and aging-associated skin disorders, including acne vulgaris, atopic dermatitis, psoriasis, rosacea, and premature skin aging [5,6,7]. Importantly, gut–skin communication is not mediated through a single pathway but rather through interconnected immune, endocrine, metabolic, and neuronal signaling networks [5,6,7]. Gut-derived metabolites, inflammatory mediators, microbial components, and oxidative stress-associated molecules may enter systemic circulation and subsequently influence skin physiological homeostasis [11,28,29]. Accordingly, modulation of the gut microbiota and microbial metabolic activity is being investigated as a potential strategy for supporting skin resilience and healthy skin aging [5,6,7,28].

### 3.2. Gut Microbiota and Intestinal Barrier Function

The intestinal barrier plays a fundamental role in maintaining host homeostasis by regulating selective nutrient transport while preventing the translocation of harmful microbial products and toxins into systemic circulation [69,70]. This barrier consists of multiple interconnected components, including epithelial cells, tight junction proteins, mucus layers, antimicrobial peptides, and immune cells, all of which are strongly influenced by the gut microbial composition and metabolic activity [69,70,71]. Healthy gut microbiota contributes to intestinal barrier integrity through the production of beneficial microbial metabolites, particularly SCFAs, such as butyrate, which serve as a major energy source for colonocytes and support epithelial homeostasis [28,29]. In contrast, gut dysbiosis may impair epithelial barrier function, increase intestinal permeability, and facilitate the systemic dissemination of lipopolysaccharides (LPS), microbial fragments, and proinflammatory mediators [71,72]. This phenomenon, commonly referred to as “leaky gut,” has increasingly been associated with chronic inflammatory conditions and gut–skin axis dysregulation [72]. Increased intestinal permeability may contribute to systemic immune activation and oxidative imbalance, thereby influencing epidermal homeostasis and cutaneous inflammatory conditions [69,70,71,72]. Elevated levels of circulating inflammatory mediators, including tumor necrosis factor-alpha (TNF-α), interleukin-6 (IL-6), and reactive oxygen species (ROS), may disrupt keratinocyte-associated homeostasis, impair epithelial differentiation, and contribute to the deterioration of the extracellular matrix associated with skin aging [28,29]. Therefore, maintenance of intestinal barrier integrity is increasingly recognized as a critical component of gut–skin communication and microbiome-associated skin physiological regulation [28,29,69,70,71,72].

### 3.3. Immune and Inflammatory Communication Between the Gut and Skin

Both the gut and skin are highly immunologically active barrier organs that continuously interact with environmental stimuli and microbial communities [73,74,75]. Bidirectional communication between the gut and skin is coordinated through interconnected immune and inflammatory networks involving cytokines, chemokines, microbial metabolites, and immune cell trafficking [73,74,75]. Gut dysbiosis may contribute to chronic low-grade systemic inflammation through the disruption of immune homeostasis and excessive production of proinflammatory mediators. Elevated levels of circulating inflammatory cytokines, including TNF-α, interleukin-1β (IL-1β), and IL-6, are associated with impaired epidermal homeostasis, oxidative imbalance, and aging-associated cutaneous dysfunction [73,74,75]. Persistent inflammatory conditions may additionally contribute to extracellular matrix deterioration and reduced skin tissue resilience [73]. In addition to innate immune responses, adaptive immune pathways, including T helper 17 (Th17) and regulatory T-cell (Treg)-associated balance, are increasingly recognized as critical components of gut–skin communication [74]. Altered immune homeostasis may therefore contribute to inflammatory skin disorders such as psoriasis and atopic dermatitis [73,74]. Emerging evidence further suggests that microbiome-derived metabolites may influence immune-associated signaling networks involved in inflammatory regulation, epithelial homeostasis, and oxidative stress-associated responses [11,28,29]. Taken together, these findings highlight the importance of immune communication in mediating gut microbiota-associated effects on skin physiology and aging [73,74,75].

### 3.4. Microbial Metabolites as Mediators of Gut–Skin Communication

Gut microbial metabolites are increasingly recognized as key mediators that link dietary factors, intestinal homeostasis, immune regulation, and skin physiology [11,35,76]. Beyond alterations in microbial composition alone, the metabolic activity of the gut microbiota may substantially influence gut–skin communication and microbiome-associated physiological regulation [11,35]. Various microbiome-derived metabolites, including SCFAs, tryptophan-derived indoles, phenolic metabolites, urolithins, bile acid-associated metabolites, and lipid-derived signaling molecules, have been implicated in epithelial homeostasis, immune communication, and microbiome–host metabolic interactions relevant to skin physiology [28,35,76]. Emerging evidence suggests that natural dietary bioactives may influence the generation of these metabolites through modulation of the gut microbial composition and microbial metabolic activity [8,11,76]. As a result, microbiome-derived metabolites have been increasingly investigated as potential mechanistic mediators linked to diet, the gut microbiota, and skin physiological homeostasis [35,76].

### 3.5. Biological Processes Linking the Gut–Skin Axis to Skin Aging

Skin aging is a multifactorial biological process influenced by intrinsic aging, environmental stressors, immune dysregulation, oxidative damage, and microbial interactions [1,2,3,77,78,79]. The gut microbiota and microbiome-derived metabolites may influence oxidative balance, inflammatory homeostasis, mitochondrial-associated physiology, and epidermal barrier integrity, thereby contributing to aging-associated cutaneous alterations [11,28,76,77,78]. The following subsections provide a concise biological context for these aging-associated processes, while their detailed microbiome-related mechanisms are examined separately in Section 6 to avoid duplication and maintain a clear progression from biological basis to mechanistic interpretation.

#### 3.5.1. Oxidative Stress and Mitochondrial Dysfunction

Oxidative stress is widely recognized as a major contributor to skin aging and epidermal dysfunction [79,80,81]. Excessive production of ROS induced by ultraviolet radiation, pollution, metabolic imbalance, and chronic inflammatory conditions can damage cellular lipids, proteins, and DNA and is associated with impaired epidermal homeostasis and extracellular matrix deterioration [79,81]. Mitochondrial dysfunction is closely linked to these processes because mitochondria play central roles in cellular energy metabolism, redox regulation, and stress adaptation [77,78,80].

In the context of the gut–skin axis, emerging evidence suggests that alterations in gut microbial composition and microbial metabolite production may influence systemic oxidative balance and mitochondrial-associated physiological regulation through interconnected metabolic and immune pathways [11,76,80]. However, the direct contribution of these microbiome-associated pathways to human skin aging remains incompletely established. The specific mechanisms linking microbial metabolism, oxidative stress, and mitochondrial dysfunction are discussed in greater detail in Section 6.1 and Section 6.4.

#### 3.5.2. Inflammaging and Chronic Low-Grade Inflammation

Chronic low-grade inflammation, commonly referred to as “inflammaging,” is increasingly recognized as a hallmark of systemic and skin aging [82,83]. In the context of the gut–skin axis, dysbiosis-associated immune imbalance and increased intestinal permeability may contribute to persistent inflammatory conditions, as discussed in Section 3.3 [73,74,75,76]. Such sustained inflammatory signaling is associated with oxidative imbalance, epidermal dysfunction, extracellular matrix deterioration, and reduced skin resilience [73,77,82,83]. These observations suggest that chronic inflammatory regulation represents an important interface between altered gut microbial homeostasis and aging-associated skin physiology [76,82]. The specific microbiome-associated mechanisms underlying inflammation and chronic inflammatory signaling are discussed in greater detail in Section 6.2.

#### 3.5.3. Skin Barrier Dysfunction and Epidermal Homeostasis

The skin barrier serves as a critical protective interface that limits excessive water loss and protects against environmental insults, pathogens, allergens, and chemical stressors [84,85]. Disruption of epidermal barrier integrity is associated with increased transepidermal water loss (TEWL), skin sensitivity, impaired epidermal resilience, and aging-associated cutaneous dysfunction [84,85]. Emerging evidence suggests that alterations in gut microbial composition and microbial metabolite production may be associated with epidermal homeostasis and barrier-related physiological regulation through gut–skin communication [4,6,76]. However, the direct contribution of gut microbiota-derived signals to human skin barrier function remains incompletely established. The specific mechanisms linking microbial metabolism to keratinocyte differentiation, epithelial signaling, lipid homeostasis, and skin barrier integrity are discussed in greater detail in Section 6.3.

#### 3.5.4. Cellular Senescence and Extracellular Matrix Degradation

**Figure 1:**
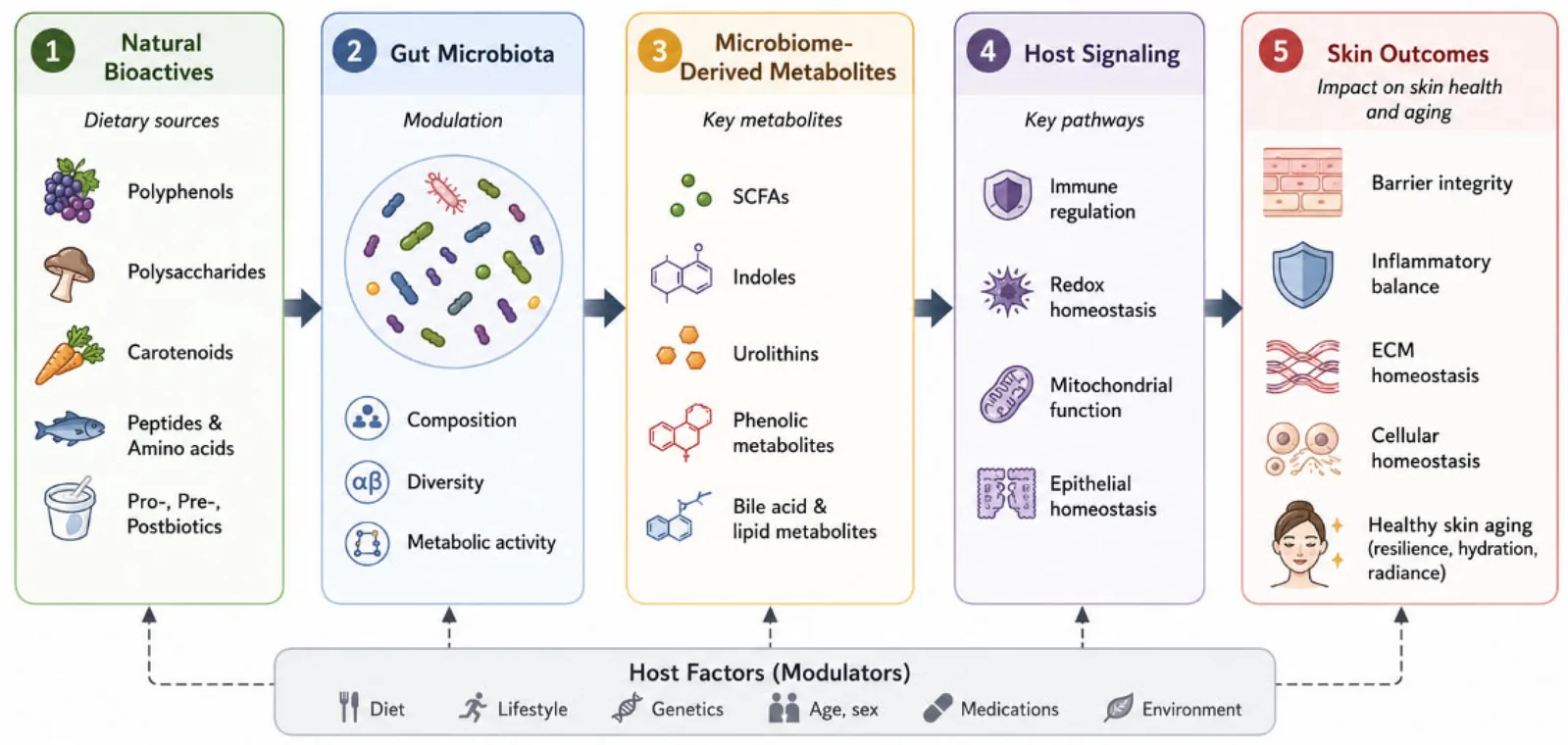
Proposed systems-level framework linking dietary bioactives, gut microbial metabolism, microbiome-derived metabolites, and skin physiology. Dietary bioactives may influence gut microbial composition and metabolic activity, thereby modifying the production of metabolites such as SCFAs, indole derivatives, phenolic metabolites, urolithins, and bile acid-associated metabolites. These metabolites may interact with immune, metabolic, redox, mitochondrial, and epithelial signaling pathways relevant to skin homeostasis and healthy skin aging.

Cellular senescence is characterized by irreversible cell cycle arrest accompanied by altered metabolic activity and the development of a senescence-associated secretory phenotype (SASP), which may contribute to chronic inflammatory signaling and impaired tissue homeostasis [86,87]. In aging skin, senescence-associated changes together with persistent oxidative and inflammatory stress are linked to extracellular matrix deterioration, collagen fragmentation, reduced skin elasticity, and impaired tissue resilience [77,79,86]. Emerging evidence suggests that altered gut microbial composition and microbial metabolite production may be associated with cellular and tissue homeostasis relevant to skin aging; however, direct causal evidence linking gut microbial alterations to cutaneous senescence and extracellular matrix degradation in humans remains limited [11,76,86]. The microbiome-associated mechanisms involving mitochondrial dysfunction, cellular senescence, and extracellular matrix remodeling are discussed in greater detail in Section 6.4 and Section 6.5. The interconnected relationships among natural bioactive compounds, the gut microbiota, microbiome-derived metabolites, and skin physiological regulation associated with the gut–skin axis are summarized in Figure 1.

## 4. Natural Bioactives as Modulators of the Gut–Skin Axis

In this review, “microbiome-targeted” approaches refer to dietary, probiotic, postbiotic, or related interventions intended to modify microbial composition, microbial metabolic activity, or microbiome-derived metabolite production in ways potentially relevant to host and skin physiology.

### 4.1. Polyphenols and Phenolic Compounds

Polyphenols represent among the most extensively studied classes of natural bioactive compounds associated with the modulation of the gut microbiota and physiological regulation of the skin. These compounds are widely distributed in fruits, vegetables, tea, cocoa, medicinal herbs, and functional foods and include flavonoids, phenolic acids, stilbenes, lignans, and tannins. Accumulating evidence suggests that the biological effects of dietary polyphenols can be substantially shaped by gut microbial metabolism, resulting in the generation of bioactive microbial metabolites with potential systemic physiological relevance [47,48,49]. Many polyphenols exhibit relatively limited absorption in the small intestine and undergo extensive microbial biotransformation in the colon [47,48,49]. The gut microbiota-mediated conversion of ellagitannins and ellagic acid into urolithins has attracted considerable attention in microbiome and nutritional research [40,41]. In particular, urolithin A is increasingly recognized as a representative microbial metabolite associated with healthy skin aging and microbiome-associated tissue resilience [40,41]. In addition to urolithins, the microbial metabolism of flavonoids such as catechins, quercetin, anthocyanins, and procyanidins can generate smaller phenolic derivatives associated with antioxidant-associated and skin-supportive physiological activities [47,48,49]. Furthermore, dietary polyphenols may selectively promote beneficial bacterial populations, including Lactobacillus and Bifidobacterium, while suppressing pathogenic microorganisms associated with gut dysbiosis. These bidirectional interactions between dietary polyphenols and the gut microbiota highlight the importance of gut microbial metabolism and host–microbiome cometabolism in microbiome-targeted nutricosmetic and skin health applications [47,48,49].

### 4.2. Polysaccharides and Dietary Fiber

Dietary polysaccharides and fermentable fibers are key modulators of gut microbial ecology and microbial metabolic activity. Natural polysaccharides derived from mushrooms, seaweeds, cereals, medicinal plants, and functional foods may function as prebiotic substrates that selectively stimulate beneficial microbial populations and promote the production of microbial metabolites involved in gut–skin communication [30,88,89,90]. Among these compounds, mushroom-derived polysaccharides, including β-glucans and heteropolysaccharides, have attracted increasing attention because of their microbiome-modulating and functional properties [91]. These polysaccharides may increase the abundance of SCFA-producing bacterial populations while supporting microbial diversity and intestinal homeostasis [30,88,89,90,91]. Microbial fermentation of dietary fibers and polysaccharides generates metabolites such as acetate, propionate, and butyrate, which are increasingly recognized as important mediators of gut–skin communication and epithelial physiological regulation [28,29,30]. Emerging evidence further suggests that dietary fibers and polysaccharides may influence skin hydration, epidermal homeostasis, and inflammatory skin conditions through the regulation of gut microbial diversity and microbial metabolite production. Natural polysaccharides capable of enhancing SCFA production may therefore contribute indirectly to epidermal resilience and healthy skin aging through microbiome-mediated mechanisms. Taken together, these findings provide a mechanistic basis for further investigations of polysaccharide-rich functional foods and mushroom-derived bioactive compounds in microbiome-targeted nutricosmetic applications [30,88,89,90,91].

### 4.3. Carotenoids and Lipophilic Bioactives

Carotenoids and other lipophilic bioactives have attracted considerable attention in nutricosmetic and skin health research because of their photoprotective and skin-supportive properties. Naturally occurring carotenoids such as astaxanthin, lycopene, β-carotene, lutein, and zeaxanthin are widely distributed in marine organisms, fruits, vegetables, and microalgae and are associated with the maintenance of epidermal physiological function and protection against environmental stress-associated skin damage [92,93,94]. Dietary carotenoids are being increasingly incorporated into oral nutricosmetic formulations because of their potential role in supporting skin hydration, elasticity, and overall skin appearance. Among these compounds, astaxanthin has attracted particular attention because of its strong antioxidant-associated potential and its relationship with skin resilience and healthy skin aging [93]. Lycopene and β-carotene have been linked to protection against ultraviolet (UV)-associated skin damage and maintenance of epidermal homeostasis [92,94]. Recent evidence suggests that the gut microbiota may influence the absorption, metabolism, and systemic bioavailability of lipophilic bioactive compounds [95,96]. Conversely, dietary carotenoids and lipid-soluble compounds may also modulate the gut microbial composition and microbial metabolic activity. These bidirectional interactions indicate that gut microbial ecology may contribute substantially to the physiological efficacy of carotenoid-based nutricosmetic interventions. In addition to carotenoids, omega-3 polyunsaturated fatty acids (PUFAs) have gained increasing attention as microbiome-responsive bioactive compounds that are relevant to skin physiology and aging [59,60]. Omega-3 fatty acids may modulate the gut microbial composition, intestinal homeostasis, and microbial metabolic activity associated with skin physiological regulation. Furthermore, host–microbiome interactions involving dietary lipids may contribute to the generation of lipid-associated microbial metabolites involved in epidermal homeostasis and skin resilience [59,60,61].

Taken together, carotenoids and lipophilic bioactive compounds warrant further investigation as potential components of microbiome-targeted nutricosmetic strategies, particularly with respect to their interactions with gut microbial composition and metabolic pathways [59,60,61,92,93,94,95,96].

### 4.4. Peptides, Fatty Acids, and Fermented Bioactives

Bioactive peptides, fatty acids, and fermented food-derived compounds are being investigated as potential components of microbiome-targeted nutricosmetic interventions because of their potential roles in supporting skin homeostasis and healthy skin aging [97,98,99]. Current evidence suggests that these bioactive compounds may have beneficial effects on skin physiology not only through direct nutritional functions but also through modulation of the gut microbial composition and microbial metabolic activity [8,11,97,100]. Among these compounds, collagen-derived peptides are widely utilized in oral beauty supplements and nutricosmetic formulations [101,102]. Hydrolyzed collagen peptides have been associated with improved skin hydration, elasticity, and dermal structural support [101,102,103]. Recent studies additionally suggest that the gut microbiota may influence peptide metabolism, absorption, and systemic bioavailability, highlighting the importance of host–microbiome interactions in collagen-associated interventions [11,97,104].

Fermented foods and fermentation-derived bioactive compounds have also attracted increasing attention because of their ability to modulate gut microbial ecology and generate biologically active metabolites [63,105,106]. Fermentation processes may increase the bioavailability of natural compounds and generate diverse small molecules, including peptides, organic acids, amino acid derivatives, and postbiotic metabolites, that are involved in the physiological homeostasis of the gut and skin [63,105,107]. In addition to peptides, dietary fatty acids, including omega-3 PUFAs, have attracted increasing interest as microbiome-responsive bioactive compounds relevant to skin physiology. These fatty acids may influence the gut microbial composition, intestinal homeostasis, and microbial metabolic activity linked to epidermal physiological regulation [59,60,61,108]. Furthermore, host–microbiome interactions involving dietary lipids may contribute to the production of lipid-associated metabolites involved in epidermal resilience and skin homeostasis [61,108]. Fermented functional foods containing probiotics, prebiotics, and postbiotic metabolites are also being investigated within “beauty-from-within” approaches for supporting skin appearance and physiological function [9,10,63,109]. These interventions may influence gut–skin communication through the modulation of microbial diversity and microbial metabolite production [5,6,63,109].

Collectively, peptides, fatty acids, and fermented bioactive compounds represent important components of microbiome-targeted nutricosmetic strategies aimed at supporting epidermal homeostasis and healthy skin aging through interconnected gut microbiota-associated pathways [63,97,98,99,100,101,102,103,104,105,106,107,108,109].

### 4.5. Probiotics, Postbiotics, and Synbiotic Approaches

Probiotics, postbiotics, and synbiotic formulations are being increasingly investigated as microbiome-targeted strategies for supporting skin health and nutricosmetic applications [9,10,110]. These approaches primarily aim to modulate the gut microbial composition, microbial metabolic activity, and gut–skin communication pathways involved in epidermal physiological regulation and healthy skin aging [5,6,9]. Probiotics are defined as live microorganisms that confer health benefits to the host when they are administered in adequate amounts [20,111]. Various probiotic strains, particularly Lactobacillus and Bifidobacterium species, have been associated with the maintenance of skin hydration, epidermal homeostasis, and skin physiological resilience [20,111]. Clinical and experimental studies further suggest that probiotic supplementation may support skin physiology through the regulation of gut microbial balance and microbial metabolic activity associated with gut–skin communication. In addition to live probiotics, increasing attention has been given to postbiotics, which refer to nonviable microbial cells, microbial metabolites, and fermentation-derived bioactive compounds capable of exerting physiological benefits [10,64]. Postbiotics may include SCFAs, peptides, exopolysaccharides, cell wall fragments, and other microbial metabolites involved in the physiological homeostasis of the gut and skin [10,29]. Compared with live probiotics, postbiotics may offer advantages in terms of formulation stability, safety, storage, and industrial applicability for cosmetic and nutricosmetic products [10,64]. Synbiotic approaches combining probiotics with prebiotic substrates have also attracted increasing interest because of their potential synergistic effects on gut microbial ecology and microbial metabolite production. Natural bioactives such as dietary fibers, mushroom polysaccharides, and polyphenols may function as prebiotic substrates that selectively stimulate beneficial microbial populations and support the generation of microbial metabolites relevant to skin physiology [47,48,89,110]. Emerging evidence suggests that microbiome-targeted interventions may support epidermal homeostasis through the modulation of gut microbial diversity, microbial metabolic activity, and microbiome-associated homeostasis [110,111]. Furthermore, personalized probiotic and synbiotic interventions based on individual microbiome characteristics and microbial metabolic responsiveness are being explored as potential personalized approaches in precision nutricosmetic research [110,112,113,114]. Recent advances in microbiome science have accelerated interest in integrated topical and oral approaches that target both the gut microbiota and skin microbiota simultaneously. Such combined strategies may warrant further investigation because of their potential to support skin physiological resilience and healthy skin aging through interconnected gut–skin microbial pathways [4,5,6,110]. Overall, probiotics, postbiotics, and synbiotic formulations represent active areas of investigation in microbiome-centered nutricosmetics; however, their clinical relevance for personalized skin health management requires further validation [20,64,110,111].

## 5. Microbial Metabolites Linking Diet, Gut Microbiota, and Skin Health

Recent advances in microbiome research have shifted attention from microbial composition alone toward the functional significance of gut microbial metabolites [11,13,14]. Recent findings indicate that the gut microbiota functions as a dynamic metabolic regulator capable of converting dietary compounds into biologically active molecules that influence immune homeostasis, epithelial signaling, oxidative balance, and systemic physiological regulation [11,14,76]. Consequently, microbial metabolites are being increasingly recognized as candidate mechanistic mediators that link diet, the gut microbiota, and gut–skin communication [13,14].

Various microbial metabolites, including SCFAs, tryptophan-derived indoles, phenolic metabolites, urolithins, bile acid derivatives, and lipid-associated signaling molecules, may regulate inflammatory signaling, epithelial homeostasis, mitochondrial-associated pathways, and host–microbiome communication [14,28,35,40]. Importantly, the generation and biological activity of these metabolites are strongly influenced by dietary composition, microbial diversity, host metabolism, and environmental exposure [11,14]. Therefore, understanding microbial metabolic pathways is essential for elucidating gut–skin communication and developing microbiome-targeted nutricosmetic strategies [14].

Importantly, the strength of evidence supporting these metabolite-mediated gut–skin pathways vary substantially across experimental systems. Mechanistic evidence is largely derived from *in vitro* studies and animal models, which are valuable for identifying molecular targets and biological pathways but do not necessarily establish physiological relevance in humans. Observational human studies provide clinically relevant associations between microbiome characteristics, microbial metabolites, and skin phenotypes but remain susceptible to confounding and cannot establish causality. Human interventional studies provide greater translational relevance; however, the current evidence remains limited by relatively small sample sizes, heterogeneous interventions, variable endpoints, and insufficient replication across independent populations. Accordingly, mechanistic plausibility, human association, and clinically demonstrated efficacy are distinguished throughout this review, and conclusions are interpreted according to the level and consistency of the available evidence.

### 5.1. SCFAs

SCFAs, primarily acetate, propionate, and butyrate, are among the most extensively studied gut microbial metabolites associated with immune regulation and epithelial homeostasis [28,29,31,32]. These metabolites are generated through microbial fermentation of dietary fibers, resistant starches, and polysaccharides within the colon and are increasingly recognized as critical signaling mediators involved in gut–skin communication [28,30,31]. Among SCFAs, butyrate has attracted particular attention because of its role in intestinal epithelial homeostasis and immune regulation. Butyrate functions as a major energy source for colonocytes and contributes to the maintenance of epithelial integrity and mucosal homeostasis [31,32,33]. Preservation of intestinal homeostasis may subsequently influence systemic inflammatory conditions associated with gut–skin interactions [29,31,32]. Experimental evidence indicates that SCFAs can regulate diverse immune and metabolic signaling pathways relevant to epithelial and systemic physiological regulation. These metabolites may influence nuclear factor kappa B (NF-κB)-associated inflammatory signaling, regulatory T-cell (Treg) differentiation, cytokine-associated immune pathways, and AMP-activated protein kinase (AMPK)-associated metabolic signaling [31,32,33,34]. Emerging evidence further suggests that SCFAs may contribute to oxidative stress-associated regulation through pathways linked to nuclear factor erythroid 2-related factor 2 (Nrf2) [33,34]. Recent studies have also implicated SCFAs in epithelial differentiation, barrier-associated homeostasis, and oxidative stress-associated physiological regulation. Accordingly, alterations in SCFA production associated with gut dysbiosis may influence gut–skin communication and systemic homeostasis [29,34]. Conversely, dietary interventions capable of enhancing SCFA-producing microbial populations warrant further investigation because of their potential relevance to microbiome-targeted nutricosmetic strategies [30,34].

### 5.2. Tryptophan Metabolites and Indole Signaling

The gut microbial metabolism of tryptophan generates diverse indole derivatives that are increasingly recognized as key mediators of epithelial homeostasis, immune regulation, and host–microbiome communication. Unlike host-mediated kynurenine metabolism, microbial tryptophan metabolites may directly influence epithelial and immune-associated signaling pathways involved in gut–skin communication [35,36,37,38].

Major microbial tryptophan metabolites include indole-3-acetic acid, indole-3-propionic acid, indole aldehydes, tryptamine, and skatole, which are generated through microbial enzymatic conversion of dietary tryptophan [36,37,38]. These metabolites are increasingly recognized as endogenous ligands of the aryl hydrocarbon receptor (AhR), a ligand-activated transcription factor involved in epithelial signaling, immune homeostasis, and barrier-associated regulation [36,37]. AhR signaling is considered a central component of epithelial and immune communication associated with the gut–skin axis. Activation of AhR pathways may regulate keratinocyte differentiation, antimicrobial peptide expression, epithelial resilience, and oxidative stress-associated responses. Conversely, dysregulation of AhR signaling has been associated with altered epithelial homeostasis and chronic inflammatory conditions linked to skin dysfunction [36,37]. Emerging evidence suggests that indole metabolites may influence inflammatory and oxidative stress-associated signaling networks involved in host–microbiome interactions [35,36,38]. Several indole derivatives have been implicated in cytokine-associated immune regulation, epithelial stress responses, and mitochondrial-associated physiological signaling, highlighting their importance as microbiome-derived signaling mediators [36,37,38]. Importantly, natural dietary bioactives may influence microbial tryptophan metabolism and indole production through the modulation of gut microbial composition and metabolic activity [36,47,48]. Polyphenols, dietary fibers, and fermented bioactive compounds may alter the abundance of indole-producing microbial populations, thereby indirectly influencing AhR-associated signaling pathways relevant to gut–skin communication [30,36,47,63]. Recent advances in microbiome and metabolomics research have further highlighted the therapeutic potential of targeting indole-associated pathways in microbiome-targeted nutricosmetic interventions [14,36,37,38]. Collectively, these findings support the potential role of microbial tryptophan metabolites as mechanistic mediators linking diet, the gut microbiota, epithelial signaling, and skin physiological homeostasis [36,37,38].

### 5.3. Phenolic Metabolites and Urolithins

Dietary polyphenols undergo extensive microbial biotransformation within the gastrointestinal tract, resulting in the generation of smaller phenolic metabolites with enhanced systemic bioavailability and biological activity [40,47,48,49]. A growing body of evidence indicates that the physiological effects of many dietary phytochemicals depend not only on the parent compounds themselves but also on metabolites generated through host–microbiome cometabolism [11,40,47,48,49]. Among these metabolites, urolithins generated from ellagitannins and ellagic acid have attracted substantial attention because of their links to metabolic signaling, mitochondrial regulation, and aging biology [40,41,42]. Urolithin A, in particular, has emerged as a representative microbiome-derived metabolite associated with mitophagy-related signaling and mitochondrial quality control. These properties provide a mechanistic rationale for investigating urolithin A in the context of skin aging, where mitochondrial dysfunction and oxidative imbalance are key contributors to age-associated cutaneous deterioration [41,43]. However, direct skin-specific clinical evidence for urolithin A remains limited. Emerging studies suggest that urolithin A may influence AMPK-associated pathways, autophagy-related signaling, and Nrf2-associated antioxidant responses [41,43]. These pathways are closely related to cellular stress adaptation, mitochondrial homeostasis, and host–microbiome metabolic communication [43]. Thus, urolithin A provides a useful example of how gut microbial metabolism can convert poorly absorbed dietary phytochemicals into biologically active metabolites with systemic relevance [41,43]. In addition to urolithins, the microbial metabolism of flavonoids, including catechins, quercetin, anthocyanins, and procyanidins, can generate diverse phenolic derivatives associated with inflammatory balance, oxidative stress regulation, and extracellular matrix-related physiological processes. Several phenolic metabolites have also been linked to the modulation of NF-κB-associated inflammatory pathways and matrix metalloproteinase (MMP)-related signaling, both of which are relevant to skin aging-associated tissue remodeling [47,48,49]. Importantly, interindividual variability in gut microbial composition may substantially influence the generation and efficacy of phenolic metabolites. The concept of “metabotypes,” which refers to individual differences in microbial metabolic capacity, has gained increasing attention in microbiome and nutrition research [42,44,45,46]. Variability in urolithin-producing capacity among individuals may partially explain the inconsistent responses to dietary polyphenol interventions and highlights the growing importance of precision microbiome-targeted nutricosmetic strategies [44,45,46]. Recent advances in metabolomics and microbiome profiling technologies have further expanded the understanding of host–microbiome cometabolism and its relevance to skin physiology [14,42,43,44,45,46]. Collectively, these findings support the concept that microbiome-derived phenolic metabolites function as critical mechanistic mediators linking natural dietary bioactive compounds, microbial metabolic signaling, mitochondrial regulation, and gut–skin communication [40,41,42,43,44,45,46].

### 5.4. Bile Acid and Lipid-Derived Metabolites

The gut microbiota participates extensively in bile acid transformation and lipid metabolism, generating diverse secondary bile acids and lipid-derived metabolites involved in host–microbiome metabolic communication and epithelial homeostasis [54,55,56]. Emerging evidence suggests that these metabolic pathways may contribute to the gut–skin communication and systemic physiological regulation associated with skin health and aging [14,54].

Primary bile acids synthesized in the liver undergo extensive microbial modification within the gastrointestinal tract through deconjugation, dehydroxylation, and oxidation reactions, resulting in the formation of secondary bile acids. These metabolites may function as signaling molecules through the activation of receptors, including farnesoid X receptor (FXR) and Takeda G protein-coupled receptor 5 (TGR5), both of which are increasingly recognized as key regulators of immune balance, metabolic signaling, and epithelial physiological homeostasis [54,56]. FXR- and TGR5-associated pathways have been implicated in inflammatory regulation, oxidative stress-associated responses, lipid metabolism, and epithelial signaling [54]. Dysregulation of bile acid metabolism has been associated with altered metabolic balance, impaired epithelial homeostasis, and chronic inflammatory conditions relevant to gut–skin communication [54,56]. In addition to bile acids, the gut microbiota interacts with dietary lipids and fatty acids to generate bioactive lipid mediators involved in host–microbiome metabolic interactions [57,59,60,61]. These lipid-associated metabolites may regulate keratinocyte-associated signaling, epidermal lipid balance, cytokine-associated pathways, and epithelial physiological regulation associated with skin homeostasis [57,58]. Recent evidence further suggests that omega-3 PUFAs and lipid-derived microbial metabolites may influence inflammatory signaling and oxidative stress-associated pathways linked to skin physiology and aging [58,59,60,61]. Host–microbiome interactions involving lipid metabolism may additionally contribute to the production of specialized proresolving mediators associated with inflammation resolution and tissue homeostasis [57,58]. Although bile acid- and lipid-derived microbial metabolites remain less extensively characterized than SCFAs and indole metabolites, expanding evidence highlights their importance in metabolic signaling and gut–skin communication [54,55,56,57,58]. Taken together, these findings support the concept that microbial metabolic networks extend beyond classical microbial metabolites and involve complex host–microbiome interactions relevant to skin physiology and healthy skin aging [14,54,55,56,57,58].

### 5.5. Integrated Microbial Metabolic Networks in Skin Health

Microbial metabolites involved in gut–skin communication do not function independently but operate within interconnected metabolic and signaling networks. SCFAs, indole derivatives, phenolic metabolites, urolithins, bile acid derivatives, and lipid-associated signaling molecules collectively contribute to immune regulation, epithelial signaling, oxidative stress-associated responses, mitochondrial regulation, and host–microbiome metabolic communication [28,35,40,54,57]. Importantly, these metabolic interactions are dynamically shaped by dietary composition, gut microbial diversity, host genetics, environmental exposures, lifestyle-associated factors, and aging-related physiological changes [11,13,14]. Therefore, the biological effects of natural dietary bioactives targeting the gut–skin axis may vary substantially among individuals, highlighting the importance of microbiome-responsive and personalized approaches in skin health management [46,112,113,115].

Recent advances in metabolomics, metagenomics, transcriptomics, and systems biology have accelerated the understanding of microbial metabolic networks involved in gut–skin communication [14,15,113,116]. Multiomics approaches have increasingly revealed that microbial metabolites may simultaneously regulate interconnected signaling pathways, including Nrf2-associated antioxidant signaling, NF-κB-associated inflammatory pathways, AhR-associated epithelial signaling, AMPK-associated metabolic regulation, and mitochondrial-associated signaling networks [113,116]. Emerging evidence additionally suggests that host–microbiome cometabolism plays important roles in determining the generation, bioavailability, and biological activity of microbial metabolites [11,15,46,115]. Interindividual differences in microbial metabolic capacity, commonly referred to as “metabotypes,” may significantly influence metabolic responsiveness and subsequent physiological outcomes [15,46,113]. These findings provide a rationale for personalized nutricosmetic approaches based on individual microbiome characteristics and metabolic responsiveness [112,113,114,115]. Furthermore, microbial metabolites may interact synergistically or antagonistically within complex metabolic ecosystems rather than functioning through isolated biochemical pathways [14,15,116]. Such interactions may collectively influence immune signaling, epithelial homeostasis, oxidative stress regulation, mitochondrial-associated physiology, and host–microbiome communication relevant to gut–skin interactions [14,116].

**Figure 2:**
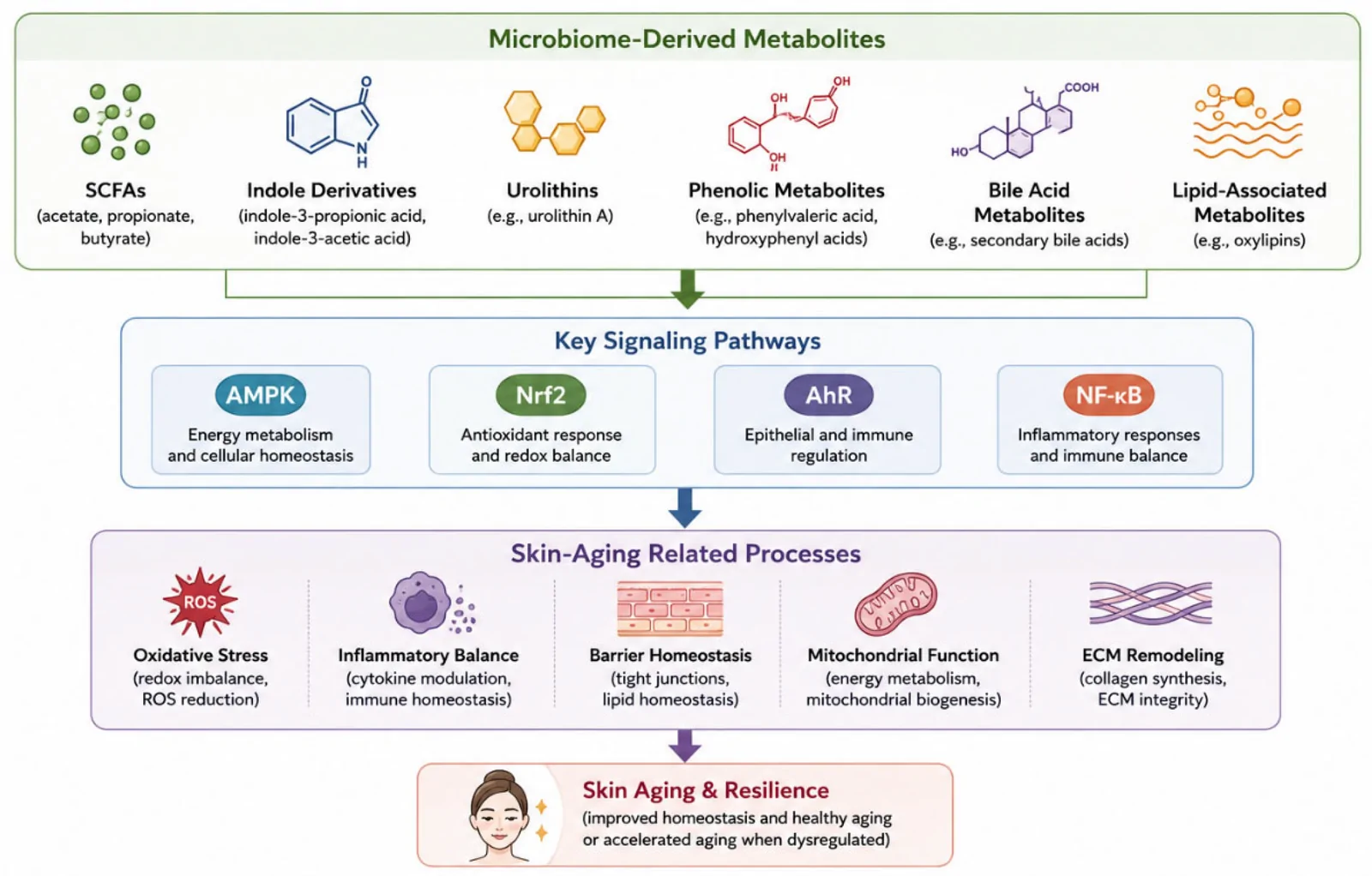
Major microbiome-derived metabolite signaling pathways relevant to gut–skin communication. Microbial metabolites, including SCFAs, indole derivatives, phenolic metabolites, urolithins, and bile acid-associated metabolites, may interact with AMPK-, Nrf2-, AhR-, and NF-κB-associated pathways. These signaling networks are involved in redox and inflammatory regulation, epithelial homeostasis, mitochondrial function, extracellular matrix remodeling, and skin aging-associated physiology.

Taken together, the current evidence supports the concept that the gut microbiota functions as a dynamic metabolic regulator capable of transforming natural dietary bioactive compounds into biologically active metabolites involved in interconnected microbiome–immune–metabolic signaling networks [11,13,14,15,115,116]. These insights provide a mechanistic basis for further investigations of personalized nutricosmetic strategies that account for individual microbial and metabolic characteristics. Interconnected microbial metabolite signaling pathways involved in gut–skin communication and skin aging-associated physiological regulation are illustrated in Figure 2.

Major microbiome-derived metabolites and their mechanistic relevance to gut–skin communication and skin aging-associated physiological regulation are summarized in Table 1.

## 6. Mechanistic Pathways in Skin Health and Aging

Skin aging is a multifactorial biological process driven by complex interactions among oxidative stress, chronic inflammation, mitochondrial dysfunction, extracellular matrix deterioration, and epidermal barrier impairment [1,2,3,77,78,79]. Emerging data suggest that the gut microbiota and microbial metabolites may influence these interconnected processes through immune regulation, metabolic signaling, and epithelial homeostasis associated with the gut–skin axis [11,14,28,76].

Gut microbial metabolism is increasingly recognized as an important contributor to skin resilience, tissue homeostasis, and aging-associated cutaneous dysfunction [11,14,115]. As a result, understanding the relationships between microbial metabolites and skin aging-associated physiological alterations may facilitate the development of microbiome-targeted nutricosmetic and antiaging strategies [14,15,112].

### 6.1. Oxidative Stress and Antioxidant Defense

Oxidative stress is widely recognized as a major contributor to skin aging and epidermal dysfunction [77,79,117]. Excessive production of ROS induced by ultraviolet radiation, environmental stressors, metabolic imbalance, and chronic inflammatory conditions can damage cellular lipids, proteins, and DNA within skin tissues [79,81,117]. Persistent oxidative imbalance is closely associated with impaired epidermal homeostasis, extracellular matrix deterioration, and aging-associated cutaneous dysfunction [79,117]. Emerging evidence suggests that the gut microbiota and microbial metabolites may influence systemic redox balance and oxidative stress-associated physiological regulation. Alterations in the gut microbial composition and microbial metabolic activity may affect oxidative homeostasis, epithelial resilience, and the inflammatory balance involved in gut–skin communication [14,76,117]. Cellular antioxidant defense systems, including Nrf2-associated pathways, are being increasingly recognized as critical regulators of stress adaptation and oxidative defense. Dysregulation of microbial metabolic homeostasis may therefore contribute to increased oxidative burden and impaired skin tissue resilience [117,118,119]. In addition, chronic gut dysbiosis and reduced production of beneficial microbial metabolites are associated with oxidative imbalance and impaired epithelial homeostasis. These alterations may contribute to aging-associated skin dysfunction and reduced epidermal resilience [14,28,29]. Natural bioactive compounds capable of modulating gut microbial metabolism and beneficial metabolite production may therefore support skin physiological homeostasis through the regulation of oxidative balance and epithelial resilience [40,47,117,118,119]. Collectively, these findings highlight the importance of redox homeostasis in gut–skin communication and skin aging physiology [14,117].

### 6.2. Inflammaging and Chronic Inflammatory Signaling

Chronic low-grade inflammation, commonly referred to as “inflammaging,” is increasingly recognized as a hallmark of both systemic aging and skin aging. Persistent inflammatory conditions are closely associated with epidermal dysfunction, impaired tissue homeostasis, extracellular matrix deterioration, and aging-associated cutaneous alterations [82,83,120].

Gut dysbiosis may contribute to inflammation through increased intestinal permeability, altered immune balance, and excessive production of proinflammatory mediators [28,29,69,70,71,72,73,74,75]. Elevated levels of circulating inflammatory cytokines, including TNF-α, IL-1β, and IL-6, are associated with chronic inflammatory conditions, oxidative imbalance, and altered physiological regulation of the epidermis linked to skin aging [73,82,120]. Emerging evidence suggests that microbial metabolites and host–microbiome interactions may influence inflammatory homeostasis associated with gut–skin communication. Altered microbial metabolic activity may contribute to persistent inflammatory conditions and impaired physiological balance associated with aging-related skin dysfunction [14,76]. Cellular SASP factors may further amplify inflammatory activity within skin tissues and contribute to extracellular matrix deterioration and age-associated tissue dysfunction. Persistent inflammatory conditions may additionally impair epidermal resilience and accelerate aging-associated cutaneous alterations [86,87]. Natural bioactive compounds capable of modulating gut microbial metabolism and inflammatory balance may therefore contribute to the maintenance of skin cutaneous resilience and healthy skin aging [40,42,47]. Collectively, these findings highlight the importance of chronic inflammatory regulation in gut–skin communication and aging-associated skin physiology [82,120].

### 6.3. Microbiome-Mediated Regulation of Skin Barrier and Epidermal Homeostasis

The skin barrier functions as a critical protective interface that prevents excessive water loss and protects against environmental stressors, pathogens, allergens, and chemical insults. Epidermal homeostasis is maintained through coordinated regulation of keratinocyte differentiation, tight junction integrity, lipid metabolism, antimicrobial peptide production, and epithelial homeostasis [84,85,121,122].

Disruption of skin barrier integrity is closely associated with increased skin sensitivity, impaired epidermal resilience, altered microbial colonization, and aging-associated cutaneous dysfunction. Compromised barrier function may additionally contribute to altered TEWL, chronic inflammatory conditions, oxidative imbalance, and impaired epidermal homeostasis [84,85]. Emerging evidence suggests that the gut microbiota and microbial metabolites may influence epidermal homeostasis and barrier-associated physiological regulation. Altered microbial metabolic activity and gut dysbiosis may affect keratinocyte-associated physiological pathways, epithelial differentiation, and epidermal resilience, which are involved in gut–skin communication [4,6,76]. Epithelial signaling pathways, including AhR-associated regulation and immune-associated pathways, are increasingly recognized as important contributors to epidermal homeostasis and barrier integrity. In addition, microbial metabolic activity may influence epidermal lipid balance and ceramide-associated physiological pathways involved in the maintenance of skin barrier function [36,37,121]. Conversely, chronic inflammatory conditions and oxidative imbalance associated with gut dysbiosis may impair epidermal homeostasis and contribute to chronic barrier dysfunction. Altered epithelial homeostasis may therefore accelerate skin sensitivity, reduce resilience, and aggravate aging-associated barrier impairment [73,82]. Natural bioactive compounds capable of modulating gut microbial metabolism and beneficial metabolite production may support epidermal homeostasis through interconnected gut–skin pathways [40,47,63]. Collectively, these findings provide a mechanistic rationale for further investigating microbiome-targeted nutritional interventions in relation to epidermal resilience and healthy skin aging [84,121].

### 6.4. Mitochondrial Dysfunction and Cellular Senescence

Mitochondrial dysfunction is increasingly recognized as a central hallmark of both systemic aging and skin aging [77,78,79,80]. Mitochondria play essential roles in cellular energy metabolism, redox homeostasis, calcium regulation, and stress adaptation, all of which are critical for maintaining epidermal integrity and tissue physiological balance [80,118,123]. Age-associated mitochondrial impairment may contribute to altered cellular bioenergetics, oxidative imbalance, impaired regenerative capacity, and chronic physiological dysfunction associated with skin aging [77,78,80,123].

Emerging evidence suggests that the gut microbiota and microbial metabolites may influence mitochondrial-associated physiological regulation through interconnected metabolic and immune-related pathways [11,14,15,115]. SCFAs, indole derivatives, and phenolic metabolites have been implicated in metabolic regulation associated with mitochondrial homeostasis, cellular resilience, and stress adaptation [31,32,36,41]. Among microbial metabolites, urolithin A has attracted considerable attention because of its association with mitophagy-related regulation and mitochondrial quality control [41,124]. Mitophagy, a selective autophagic process involved in the removal of damaged mitochondria, is increasingly recognized as an important component of healthy aging and tissue resilience [125,126]. Impaired mitochondrial quality control may therefore contribute to oxidative imbalance, altered cellular metabolism, and aging-associated physiological dysfunction [80,125,126]. Mitochondrial-associated signaling networks, including AMPK-associated regulation, Nrf2-associated signaling, sirtuin-related pathways, and mitochondrial biogenesis-associated regulation, are being increasingly recognized as major contributors to cellular stress adaptation and tissue homeostasis [117,118,119,123,127]. Cellular senescence is characterized by irreversible cell cycle arrest accompanied by altered metabolic activity, chronic inflammatory conditions, and the secretion of SASP factors [86,87,128,129]. Senescence-associated alterations may contribute to impaired tissue homeostasis, extracellular matrix deterioration, and aging-associated cutaneous dysfunction [128,129]. Emerging evidence additionally suggests that mitochondrial dysfunction and altered microbial metabolic regulation may contribute to senescence-associated physiological imbalance linked to skin aging [14,15,80,128]. Natural bioactive compounds capable of supporting beneficial microbial metabolite production may therefore contribute to the maintenance of cellular resilience, mitochondrial homeostasis, and tissue physiological balance associated with healthy skin aging [40,41,47,124]. Overall, these findings support the concept that microbiome–mitochondria interactions represent major components of gut–skin communication and aging-associated skin physiology [14,15,41,123,124,125,126,127,128,129]. Such interactions warrant further mechanistic and clinical investigations to determine their potential relevance to microbiome-targeted antiaging nutricosmetic strategies [40,41,124].

### 6.5. Collagen Degradation and Extracellular Matrix Remodeling

The extracellular matrix (ECM) provides structural support and mechanical integrity to skin tissues and is primarily composed of collagen, elastin, glycoproteins, and proteoglycans. Maintenance of extracellular matrix homeostasis is essential for preserving skin elasticity, firmness, hydration, and tissue resilience. Age-associated disruption of ECM integrity is considered a major contributor to visible skin aging, including wrinkle formation, dermal thinning, and reduced skin elasticity [130,131,132].

Oxidative imbalance and chronic inflammatory conditions are closely associated with collagen degradation and extracellular matrix remodeling [79,120,130,131]. Excessive production of ROS and inflammatory mediators may contribute to the activation of MMP-associated pathways involved in extracellular matrix turnover and dermal structural alteration [130,131]. Persistent inflammatory conditions may additionally impair fibroblast-associated homeostasis and collagen-associated tissue homeostasis [120,129,131,133]. Emerging evidence suggests that the gut microbiota and microbial metabolites indirectly influence extracellular matrix-associated physiological regulation through immune balance, oxidative stress-associated pathways, and host–microbiome metabolic communication. Altered microbial metabolic activity may therefore contribute to extracellular matrix imbalance and dermal physiological alterations associated with skin aging [14,15,57]. Transforming growth factor-beta (TGF-β)-associated regulation, fibroblast-associated homeostasis, and extracellular matrix-related signaling pathways are increasingly recognized as important contributors to dermal structural resilience [132,133,134]. In addition, oxidative imbalance and mitochondrial dysfunction may influence tissue structural integrity and age-associated physiological deterioration of the dermis [80,131]. Recent evidence also suggests that SASP-associated physiological conditions may amplify extracellular matrix remodeling and chronic dermal dysfunction linked to aging-related skin alterations [128,129,130,131]. Collectively, chronic inflammatory conditions and impaired mitochondrial homeostasis may indirectly contribute to reduced dermal resilience and extracellular matrix deterioration [80,120,128,131]. Importantly, natural dietary bioactives capable of modulating gut microbial metabolism and beneficial metabolite production may contribute to the maintenance of extracellular matrix homeostasis through interconnected gut–skin pathways [40,41,47,63]. Collectively, these findings provide a rationale for investigating whether microbiome-targeted nutricosmetic interventions can contribute to dermal structural resilience and healthy skin aging [40,41,97,109,124].

## 7. Nutricosmetics and Translational Applications

A growing understanding of gut–skin communication and microbial metabolic regulation has increased interest in nutricosmetic strategies targeting skin health and aging [5,14,115]. Unlike conventional cosmetic approaches, which focus primarily on topical interventions, emerging nutricosmetic research increasingly examines the systemic modulation of skin physiology through dietary bioactives, gut microbial regulation, and host–microbiome metabolic interactions [109,135].

Recent advances in microbiome science, metabolomics, and systems biology have further expanded research into microbiome-targeted functional ingredients, personalized nutritional interventions, and integrated oral–topical approaches for supporting skin homeostasis and healthy skin aging [112,113,114,116]. However, their translational application remains dependent on further mechanistic validation and well-controlled human studies [109,135].

### 7.1. Beauty-from-Within and Oral Nutricosmetics

The concept of “beauty-from-within” has gained substantial attention, as increasing evidence supports the influence of nutrition, the gut microbiota, and systemic metabolism on skin physiology and aging [109,135,136]. Oral nutricosmetics, including dietary supplements, functional foods, and microbiome-targeted formulations, are being increasingly explored as complementary or alternative approaches to conventional topical cosmetics [97,109,135].

Natural bioactives such as polyphenols, carotenoids, collagen peptides, polysaccharides, probiotics, and fermented bioactives have demonstrated potential benefits for skin hydration, elasticity, epidermal resilience, and physiological homeostasis [20,47,93,101]. Importantly, microbiome modulation has been proposed as a potential contributor to the systemic effects of some oral bioactives; however, direct evidence demonstrating microbiome mediation of clinically observed skin benefits remains limited [11,14,47]. Recent evidence suggests that oral nutricosmetic interventions may influence skin physiology through the regulation of oxidative balance, epidermal barrier integrity, extracellular matrix-associated homeostasis, and mitochondrial-associated tissue resilience. These findings support the concept that systemic nutritional modulation may contribute to the maintenance of skin physiological balance and healthy skin aging through interconnected gut–skin pathways [40,41,101,103]. Moreover, considerable interindividual variability remains an important challenge in oral nutricosmetic interventions. Differences in gut microbial composition, microbial metabolic responsiveness, dietary habits, and host physiology may substantially influence the generation and efficacy of bioactive microbial metabolites [12,46,112]. As a result, increasing attention has been given to microbiome-responsive and personalized nutricosmetic approaches capable of optimizing skin-related physiological outcomes [112,113,114]. Taken together, these findings support continued investigations of oral nutricosmetics in the context of systemic host–microbiome interactions, while direct evidence linking microbiome modulation to clinical skin benefits remains limited [109,135,136].

### 7.2. Microbiome-Targeted Functional Ingredients

Recent advances in microbiome science have accelerated interest in the development of functional ingredients specifically designed to modulate gut microbial ecology and microbial metabolite production associated with skin physiology. Unlike conventional cosmetic ingredients that primarily target localized skin effects, microbiome-targeted nutricosmetic ingredients influence systemic physiological regulation through modulation of the gut microbial composition and metabolic activity [14,137].

Dietary polyphenols, mushroom-derived polysaccharides, prebiotic fibers, fermented bioactive compounds, omega-3 fatty acids, and microbiome-responsive peptides have attracted increasing attention as potential microbiome-modulating ingredients [30,47,59,97]. These compounds may selectively stimulate beneficial microbial populations, increase the production of SCFAs and other beneficial metabolites, and support epithelial and immune homeostasis associated with gut–skin communication [28,29,30]. A growing understanding of host–microbiome cometabolism has additionally highlighted the importance of microbial biotransformation and interindividual metabolic variability during functional ingredient development [11,15,46]. Certain dietary compounds may exhibit limited efficacy in the absence of appropriate microbial metabolic activity, suggesting that microbial responsiveness represents an important determinant of nutricosmetic efficacy [12,46,112]. Moreover, substantial challenges remain regarding ingredient standardization, metabolite bioavailability, interindividual variability, and mechanistic validation. The biological activity of many microbiome-targeted ingredients may vary depending on dietary composition, microbial diversity, host metabolism, and environmental factors, complicating the prediction of physiological responses [137,138]. Collectively, microbiome-targeted ingredient development is increasingly shifting toward systems-level approaches that integrate dietary bioactives, microbial metabolism, immune regulation, and personalized nutritional strategies associated with skin health and aging [113,116,138].

### 7.3. Probiotic, Postbiotic, and Synbiotic Formulations

Probiotic, postbiotic, and synbiotic formulations are being increasingly explored as microbiome-targeted strategies for supporting skin physiology and healthy skin aging [64,110,111]. Various probiotic strains, particularly Lactobacillus and Bifidobacterium species, have demonstrated potential benefits for skin hydration, epidermal homeostasis, inflammatory balance, and barrier-associated physiological resilience [20,21]. Compared with live probiotics, postbiotics—including microbial metabolites, inactivated microbial cells, peptides, exopolysaccharides, and fermentation-derived compounds—have attracted increasing interest because of their improved formulation stability, safety, storage capacity, and scalability [10,22,64]. Postbiotic approaches may additionally provide more predictable physiological effects by directly delivering bioactive microbial products associated with gut–skin communication [22,64]. Synbiotic formulations combining probiotics with prebiotic substrates may further increase microbial metabolite production and gut microbial stability [23,110]. Emerging evidence suggests that these integrated approaches may support oxidative balance, epithelial homeostasis, inflammatory regulation, and mitochondrial-associated cutaneous resilience linked to skin aging [23,110]. Recent advances in microbiome engineering, precision fermentation technologies, and postbiotic production systems may additionally facilitate the development of next-generation microbiome-based nutricosmetics with enhanced functionality and personalized responsiveness [24,25]. However, despite growing industrial interest, robust long-term clinical evidence specifically addressing skin aging-associated outcomes is lacking for many probiotic and postbiotic interventions [26,27]. Collectively, probiotic, postbiotic, and synbiotic formulations remain active areas of translational research in microbiome-targeted nutricosmetics, although additional long-term and well-controlled clinical studies are needed to establish their efficacy for personalized skin health management [23,64,110]. Evidence supporting these microbiome-targeted approaches for human intervention is critically summarized in Section 7.4 and Table 2.

### 7.4. Clinical Evidence for Human Gut–Skin and Nutricosmetic Interventions

Although mechanistic and preclinical studies provide substantial biological plausibility for gut–skin communication, direct evidence supporting human intervention remains relatively limited and heterogeneous. Nevertheless, several randomized controlled trials have demonstrated clinically measurable skin-related effects following oral microbiome-targeted interventions. In a randomized, double-blind, placebo-controlled study, oral supplementation with *Lactobacillus plantarum* HY7714 for 12 weeks improved skin hydration, elasticity, wrinkle-related parameters, and transepidermal water loss in adults with dry and wrinkled skin [20]. Other probiotic interventions have also shown beneficial outcomes under inflammatory skin conditions. *Lactobacillus rhamnosus* SP1 supplementation improved clinical acne outcomes and modulated the skin expression of insulin signaling-related genes in adults [21], whereas multistrain probiotic formulations reduced SCORAD scores and topical corticosteroid use in children with moderate atopic dermatitis [22]. Similarly, a three-strain Lactobacillus formulation improved SCORAD scores and skin moisturization, smoothness, and inflammatory marker levels in adults with mild-to-severe atopic dermatitis [23].

Human intervention studies of oral collagen peptides and other dietary bioactives provide additional evidence that systemic nutritional interventions can influence measurable skin outcomes. Specific collagen peptides administered for 8 weeks improved skin elasticity in a randomized placebo-controlled trial [101], whereas low-molecular-weight collagen peptides administered for 12 weeks improved skin hydration, elasticity, and wrinkle-related parameters [137]. Moreover, combined supplementation with astaxanthin and collagen hydrolysate improved facial skin elasticity and transepidermal water loss and altered procollagen and matrix metalloproteinase expression in photoaged skin [138]. Importantly, however, these latter studies did not directly assess gut microbiome composition or microbiome-derived metabolites and therefore provide evidence for systemic nutricosmetic efficacy rather than direct confirmation of microbiome-mediated gut–skin mechanisms.

Overall, the available human evidence supports the clinical plausibility of microbiome-targeted and systemic oral interventions for skin health, but consistency across studies remains limited by substantial heterogeneity in intervention type, microbial strain, dose, treatment duration, participant characteristics, and clinical endpoints. Furthermore, only a limited number of clinical trials simultaneously evaluate changes in the gut microbiome or microbial metabolites together with skin-specific outcomes. Consequently, improvements in skin physiology following oral interventions should not automatically be interpreted as evidence of a causal microbiome-mediated mechanism. Future clinical studies should integrate standardized dermatological and biophysical endpoints with longitudinal gut microbiome profiling and metabolomic analyses to establish more direct causal links between microbial modulation and clinically relevant skin outcomes. To distinguish evidence supporting individual biological components from evidence supporting a complete microbiome-mediated pathway, Table 3 provides an evidence-chain appraisal of representative diet–microbiome–metabolite–skin relationships. Evidence for separate steps of a proposed pathway should not be interpreted as a demonstration of microbiome-mediated causality unless microbial alteration or biotransformation, relevant metabolite exposure, and skin-specific outcomes are linked within the same experimental or clinical framework. The appraisal therefore distinguishes clinical efficacy from evidence of microbiome mediation and identifies where the proposed causal chain remains incomplete.

### 7.5. Topical–Oral Combined Strategies

Recent evidence suggests that combined topical and oral interventions may provide complementary or potentially synergistic benefits for maintaining skin homeostasis and delaying skin aging [26,135,139]. While oral nutricosmetics primarily influence systemic metabolism and gut microbial regulation, topical formulations may directly target epidermal barrier integrity, skin microbiota, and localized oxidative stress-associated responses [109,135,139]. Integrated topical–oral approaches may therefore simultaneously modulate the gut microbiota, skin microbiota, immune balance, and epidermal physiology through interconnected pathways [139,140]. Such strategies may be particularly relevant for chronic inflammatory skin conditions, impaired barrier function, and aging-associated epidermal dysfunction [21,22,140]. Emerging microbiome-based cosmetic formulations increasingly incorporate probiotics, postbiotics, fermented bioactive compounds, and microbiome-responsive compounds designed to support both gut and skin microbial ecosystems [10,26,64,141]. In particular, growing interest has been directed toward dual microbiome-targeted approaches capable of regulating systemic microbial metabolism while simultaneously supporting local epidermal homeostasis and skin microbial balance [139,140,142]. Recent advances in delivery systems, fermentation technologies, and microbiome-responsive formulations may further facilitate the development of integrated oral–topical strategies with enhanced functionality and personalized applicability [143,144]. Collectively, integrated oral–topical approaches warrant further clinical investigation to determine whether simultaneous modulation of systemic and local microbial pathways provides additional benefits for skin health and healthy aging [139,140,141,142,143,144].

### 7.6. Challenges in Clinical Translation and Product Development

Despite growing interest in microbiome-targeted nutricosmetics, several important challenges remain regarding clinical translation, product standardization, and mechanistic validation [25,136,145]. Considerable interindividual variability in gut microbial composition and microbial metabolic responsiveness may contribute to inconsistent outcomes across clinical studies and dietary interventions [12,46,112,146]. In addition, many current studies are limited by relatively small sample sizes, heterogeneous intervention protocols, insufficient biomarker standardization, and a lack of long-term clinical validation in humans [25,146]. Variability in microbial metabolite production, dietary background, host metabolism, and environmental exposures may further complicate the interpretation of nutricosmetic efficacy and reproducibility across studies [15,46,146]. Technical challenges associated with formulation stability, microbial viability, metabolite bioavailability, storage conditions, and regulatory standardization also remain major considerations during product development [143,147]. Furthermore, the mechanisms underlying the interactions among the gut microbiota, skin microbiota, host metabolism, immune regulation, and environmental factors remain incompletely understood [14,15,116]. Another important limitation involves the relatively limited availability of skin-specific mechanistic evidence in human subjects [25,27,136,142]. Although many microbiome-targeted interventions demonstrate promising biological activities in experimental systems, causal relationships between microbial metabolites and skin aging-associated physiological outcomes remain insufficiently characterized in clinical settings [21,22,23,24,25,141,142]. Future translational efforts should therefore integrate systems biology, metabolomics, microbiome profiling, multiomics technologies, and precision nutrition approaches to improve the understanding of the underlying mechanism and optimize microbiome-responsive nutricosmetic interventions [113,114,116,146,147,148]. Greater emphasis on longitudinal human studies, standardized biomarkers, and personalized microbiome-responsive strategies may additionally facilitate the development of evidence-based next-generation nutricosmetics for healthy skin aging [145,146,147,148].

## 8. Precision Nutricosmetics and Future Perspectives

In this review, “precision nutricosmetics” refers to the emerging concept of tailoring nutritional or microbiome-responsive skin health interventions according to individual microbial, metabolic, dietary, and physiological characteristics.

Recent advances in microbiome science, systems biology, metabolomics, and computational technologies are rapidly advancing the current understanding of skin health and nutricosmetic interventions [113,114,115,116,149,150,151,152,153]. Increasing evidence suggests that interindividual variability in gut microbial composition, microbial metabolic responsiveness, and host physiology may substantially influence the efficacy of dietary bioactives and microbiome-targeted skin health strategies [12,13,42,43,44,45,46,112,149,150]. In turn, future nutricosmetic approaches are increasingly shifting from generalized interventions toward precision microbiome-responsive strategies integrating personalized nutrition, multiomics technologies, artificial intelligence (AI), and microbiome engineering [151,152,153,154,155]. These emerging approaches may facilitate the development of next-generation personalized interventions capable of optimizing skin physiology through interconnected microbiome–immune–metabolic networks [15,115,116,149,150,151,152,153]. Moreover, considerable challenges remain regarding the standardization of microbiome-associated biomarkers, reproducibility of metabolomic profiling, and interpretation of complex host–microbiome interactions [145,146,147,148,153]. Future progress in precision nutricosmetics will therefore depend on the integration of mechanistic microbiome research with clinically validated and personalized intervention strategies [112,113,114,145,146,147,148,149,150,151,152,153].

### 8.1. Personalized Gut–Skin Responses and Metabotypes

Considerable interindividual variability has been observed in responses to dietary bioactives, probiotics, and nutricosmetic interventions [42,43,44,45,46,149,150]. Accumulating evidence has demonstrated that these differences may be strongly influenced by the gut microbial composition, microbial metabolic capacity, host genetics, dietary habits, lifestyle-associated factors, and environmental exposures [13,15,112,115,149,150]. The concept of “metabotypes,” which refers to individual differences in microbial metabolic responsiveness, has gained increasing attention in microbiome and nutrition research [42,43,44,45,46]. Variability in the production of microbial metabolites such as urolithins, SCFAs, and indole derivatives may substantially influence the physiological efficacy of dietary interventions associated with skin health and aging [28,31,32,33,34,35,36,37,38,42,43,44,45,46]. These findings may partially explain the inconsistent outcomes observed in nutricosmetic clinical studies and highlight the limitations of generalized dietary recommendations [27,46,112,136,149,150]. Accordingly, precision nutricosmetic strategies based on individual microbiome characteristics, metabolic responsiveness, and physiological profiles are being investigated as potential approaches to personalized skin health management [112,113,114,149,150,151]. Future personalized interventions may integrate microbiome profiling, metabolomic biomarkers, dietary responsiveness, skin physiological assessments, and host metabolic characteristics to optimize microbiome-targeted skin health strategies [115,116,149,150,151,152,153]. Such approaches may facilitate the development of individualized interventions capable of improving skin resilience and healthy skin aging through precise microbiome-responsive regulation [112,113,114,149,150,151,152,153]. From a clinical translation perspective, however, substantial barriers remain before microbiome-based stratification can be routinely incorporated into nutricosmetic interventions. Baseline microbiome composition, microbial functional capacity, habitual diet, medication use, age, host genetics, and environmental exposures may all modify metabolic responses to the same dietary or probiotic intervention [12,13,46,112,115,146,149,150]. Consequently, conventional clinical trials that evaluate average treatment effects across heterogeneous populations may obscure biologically meaningful responder and nonresponder subgroups. Future trials should therefore incorporate prospective stratification based on baseline microbiome profiles, relevant metabotypes, and metabolic biomarkers, together with standardized dietary and skin phenotyping. Longitudinal assessment of microbiome composition, microbial metabolites, and skin-specific clinical or biophysical outcomes will also be important for determining whether baseline microbial features can reproducibly predict intervention responsiveness [15,115,145,146,149,150,151,152,153]. Such stratified study designs may help distinguish true microbiome-dependent treatment effects from interindividual variability and provide a more rigorous basis for the clinical development of precision nutricosmetic strategies.

### 8.2. Multiomics Approaches and Systems Biology

Recent advances in metagenomics, metabolomics, transcriptomics, proteomics, and lipidomics have substantially expanded the current understanding of gut–skin communication and microbiome-associated skin physiology. Multiomics approaches increasingly reveal complex interactions among dietary bioactive compounds, the gut microbiota, microbial metabolites, host metabolism, immune regulation, and aging-associated skin pathways [116,151,152].

Recent studies provide concrete examples of how multiomics approaches can move gut–skin and skin microbiome research beyond descriptive taxonomic profiling. Li et al. integrated skin microbiome and metabolome analyses to investigate the effects of skincare formulations containing prebiotics and postbiotics, demonstrating how simultaneous microbial and metabolic profiling can identify coordinated changes associated with topical intervention [152]. Similarly, Huang et al. applied multiomics analyses to atopic dermatitis and identified relationships between alterations in the skin microbiota and skin metabolite profiles, illustrating the potential of integrated microbial–metabolic datasets to characterize disease-associated skin phenotypes [153]. Although these studies focused primarily on the skin microbiome rather than direct gut–skin metabolic communication, they provide a proof-of-concept for the type of integrated analytical framework that could be extended to future gut–skin intervention studies combining the gut microbiome, circulating metabolites, and skin phenotyping data.

Integrative systems biology approaches may facilitate the identification of microbial biomarkers, metabolic signaling networks, and individualized therapeutic targets relevant to skin health and aging [15,116]. In particular, metabolomics-based profiling of microbial metabolites may provide critical insights into the mechanisms underlying interindividual variability in nutricosmetic responsiveness and microbial metabolic activity [115,151,152,153]. Network-based analyses integrating microbial composition, metabolite production, signaling pathways, and skin physiological outcomes may further improve the understanding of interconnected host–microbiome interactions associated with epidermal homeostasis and skin aging [15,115,116,151,152,153]. These approaches may help clarify the complex relationships among microbial metabolism, immune balance, oxidative stress-associated regulation, and tissue cutaneous resilience [14,116,152,153]. Despite these advances, important challenges remain regarding the standardization of omics platforms, reproducibility of large-scale datasets, biomarker validation, and interpretation of highly complex biological networks [145,146,147,148,151,152,153]. Future studies integrating longitudinal clinical data with multiomics analyses may therefore be essential for improving the mechanistic understanding and translational applicability of microbiome-targeted nutricosmetics [145,146,147,148,149,150,151,152,153]. Collectively, these emerging technologies may accelerate the development of precision microbiome-targeted interventions with enhanced mechanistic specificity, predictive capability, and personalized applicability for healthy skin aging [112,113,114,116,149,150,151,152,153].

### 8.3. Artificial Intelligence and Digital Skin Health

Artificial intelligence (AI), machine learning, and computational modeling are being increasingly integrated into microbiome and skin health research [114,149,150,154,155]. These technologies may facilitate the prediction of microbial responsiveness, identification of bioactive compound interactions, and optimization of personalized nutritional interventions associated with skin physiology and aging [114,149,150].

Clinical dermatology provides established examples of the potential of AI-assisted phenotyping. Esteva et al. demonstrated that deep neural networks could classify skin cancer images at a performance level comparable to that of dermatologists [154], whereas Liu et al. subsequently developed a deep learning system for the differential diagnosis of multiple skin diseases [155]. Although these studies were not designed to evaluate the gut–skin axis or nutricosmetic interventions, they demonstrated the feasibility of extracting clinically relevant phenotypic information from complex skin imaging datasets. In future gut–skin studies, analogous computational approaches could be integrated with microbiome, metabolomic, dietary, and longitudinal skin imaging data to investigate whether multimodal models can identify intervention-responsive phenotypes or responder subgroups. Such applications, however, remain largely prospective and require direct clinical validation in microbiome-targeted skin interventions.

In addition to dermatology, data-driven precision nutrition studies also provide relevant methodological precedents. For example, Zeevi et al. integrated dietary, clinical, and gut microbiome features to predict individualized postprandial glycemic responses [149], whereas Berry et al. demonstrated substantial interindividual variation in postprandial metabolic responses in a large-scale human cohort [150]. Although these studies did not assess skin outcomes, they illustrate how microbiome and host phenotypic data can be integrated to model individual nutritional responsiveness, providing a methodological basis for future microbiome-responsive nutricosmetic studies.

AI-assisted analysis of microbiome datasets, metabolomic profiles, dietary patterns, and skin imaging data may improve the understanding of interindividual variability in gut–skin interactions [114,149,150,151,152,153,154,155]. Predictive modeling approaches may additionally support the identification of responder and nonresponder populations during microbiome-targeted nutricosmetic interventions [149,150]. Emerging digital health technologies, including AI-assisted skin imaging systems, mobile health platforms, and wearable sensors, may further enhance personalized skin health management and longitudinal assessments of the physiological changes associated with skin aging and environmental stress responses [154]. Integration of AI-driven analytics with systems biology, microbiome profiling, and precision nutrition approaches may therefore accelerate the development of adaptive nutricosmetic platforms capable of dynamically optimizing interventions on the basis of individual physiological and microbial characteristics [114,116,149,150,151,152,153,154,155]. Nevertheless, important limitations remain regarding data interpretation, algorithm reproducibility, privacy protection, and clinical validation of AI-assisted predictive systems [145,146,147,148,154,155]. Future progress will therefore require close integration among microbiome science, computational biology, dermatology, and clinical nutrition to ensure the development of evidence-based and clinically applicable precision skin health strategies [114,145,146,147,148,154,155].

### 8.4. Microbiome Engineering and Next-Generation Nutricosmetics

Advances in microbiome engineering, synthetic biology, and precision fermentation technologies are creating new opportunities for the development of next-generation nutricosmetics targeting gut–skin communication and skin aging [156,157,158,159]. Emerging approaches aim not only to modulate microbial composition but also to selectively increase the production of beneficial microbial metabolites involved in skin physiology and tissue resilience [156,157,158].

Precision fermentation technologies may facilitate the production of postbiotics, bioactive peptides, and metabolite-enriched functional ingredients with improved stability, bioavailability, and targeted functionality [64,65,157]. Engineered probiotic strains and microbiome-responsive delivery systems may additionally provide enhanced control of microbial metabolic activity and host–microbiome interactions associated with skin health [156,158,159]. Future microbiome engineering strategies may also integrate personalized microbiome profiling, dietary modulation, metabolite-targeted interventions, and adaptive formulation technologies to optimize individual skin health outcomes [112,113,114,149,150,151,152,153,156,157,158,159]. Such approaches may ultimately support the development of precision microbiome-based cosmetic and nutricosmetic platforms capable of dynamically responding to individual physiological and microbial characteristics [152,156,157,158,159]. Moreover, important challenges remain regarding safety validation, long-term ecological stability, regulatory standardization, and ethical considerations associated with engineered microbiome-based interventions [146,147,148,158,159]. Careful evaluation of host–microbiome interactions and unintended metabolic consequences will therefore be essential for future translational applications of microbiome engineering technologies in skin health management [15,146,147,148,156,158,159].

### 8.5. Future Challenges and Research Directions

Despite rapid advances in gut–skin axis research, several important challenges remain regarding mechanistic validation, clinical standardization, and translational applicability. Current evidence is still limited by heterogeneity in study design, insufficient long-term human clinical validation, and incomplete understanding of host–microbiome interactions associated with skin physiology and aging [16,17,18,19,27,145,146,147,148].

Future research should prioritize standardized microbial biomarkers, longitudinal clinical studies, mechanistic metabolite profiling, and integrative systems biology approaches capable of clarifying causal relationships between microbial metabolism and skin physiological outcomes. Greater emphasis should additionally be placed on interindividual variability, microbial metabolic responsiveness, metabotype-associated differences, and personalized intervention strategies [18,19,151,152,153]. Another important challenge involves limited skin-specific mechanistic evidence in human subjects [16,17,27]. Although many microbiome-targeted interventions demonstrate promising biological activities in experimental systems, the precise relationships between microbial metabolites and aging-associated skin phenotypes remain incompletely understood in clinical settings [16,17,19,21,22,23,24,25,26,27]. Ethical considerations, regulatory frameworks, data privacy, commercialization challenges, and the reproducibility of AI-assisted personalized skin health technologies will also require further attention [154,155,160,161]. Expanding collaboration among microbiology, dermatology, nutrition, systems biology, computational biology, and clinical sciences may therefore be essential for advancing precision microbiome-targeted nutricosmetic research [18,19,160,161]. Collectively, future progress in microbiome science and systems-level understanding of gut–skin communication may transform current paradigms in skin health management and facilitate the development of personalized next-generation nutricosmetic strategies for healthy skin aging [18,19,149,150,151,152,153].

### 8.6. From Association to Causality: Evidence Gaps and Unresolved Questions

Despite rapid advances in gut–skin axis research, several important limitations and unresolved questions remain regarding the mechanistic interpretation and translational applicability of microbiome-targeted nutricosmetic strategies [16,17,18,19]. A central challenge in interpreting the current evidence is that support for individual components of a proposed gut–skin mechanism is sometimes extrapolated to the complete microbiome-mediated pathway. Evidence that a dietary bioactive compound modifies the gut microbial composition, that a microbiome-derived metabolite regulates a molecular pathway in experimental models, and that oral intervention improves a skin-related endpoint in separate studies does not establish that the observed clinical effect is mediated by that microbial pathway. A stronger inference of microbiome-mediated causality would require sequential evidence linking dietary exposure or intervention to microbial alteration or biotransformation, relevant metabolite production and systemic exposure, host target engagement, and standardized skin-specific outcomes within appropriately designed experimental or human studies [15,16,17,18,19]. At present, such integrated evidence remains limited, and many proposed diet–microbiome–metabolite–skin relationships should therefore be interpreted as mechanistically plausible rather than clinically established causal pathways. Although numerous microbial metabolites have demonstrated biological activity in experimental systems, skin-specific mechanistic evidence remains relatively limited for many metabolites [16,17]. Much of the current evidence is derived from *in vitro* studies, animal models, or indirect systemic observations, while well-controlled human clinical studies specifically evaluating gut microbiota-associated skin outcomes are lacking [17,19,126]. Substantial interindividual variability in gut microbial composition and microbial metabolic responsiveness further complicates the interpretation of dietary intervention studies [115,149,150]. The production of microbial metabolites such as urolithins, indole derivatives, and SCFAs may differ considerably among individuals depending on microbial diversity, dietary patterns, host genetics, age, medication use, and environmental exposures [44,45,46,115]. As a result, identical dietary interventions may produce markedly different physiological outcomes among subjects [46,149,150]. In addition, causal relationships within gut–skin communication remain incompletely understood [16,17,18,19]. Although associations between gut dysbiosis, microbial metabolites, and skin physiological alterations have been increasingly reported, the precise interactions linking microbial metabolic activity to specific aging-associated skin phenotypes remain insufficiently characterized [16,17,27]. In particular, the relative contributions of the gut microbiota, skin microbiota, immune regulation, and host metabolism require further clarification [15,17,19]. Additional challenges involve a lack of standardized biomarkers and methodological heterogeneity across microbiome studies [18,19]. Variability in sequencing approaches, metabolomics platforms, dietary intervention protocols, and skin physiological assessments complicates cross-study comparisons and reproducibility [151,152,153]. Furthermore, many microbial metabolites exhibit context-dependent and concentration-dependent biological effects, which may contribute to inconsistent findings [14,28,35,38,54,55,56,57,58].

Several unresolved issues also complicate the interpretation of the current gut–skin literature. First, whether alterations in the gut microbiome are causal drivers of skin aging-related phenotypes or predominantly reflect the shared influences of diet, age, medication use, inflammation, and other host factors remains uncertain [15,16,17,18,19,115]. Second, the clinically observed benefits of oral probiotics or nutricosmetic compounds cannot necessarily be attributed to microbiome mediation because most intervention studies do not simultaneously measure microbial composition, circulating microbial metabolites, and skin-specific outcomes [20,21,22,23,24,25,26,27]. Third, the biological relevance of individual microbial metabolites is likely to be context-dependent; the same metabolite may exert different effects according to concentration, tissue exposure, host metabolic status, and interactions with other microbial products [14,28,35,38,54,55,56,57,58]. Finally, substantial interindividual differences in microbial metabolic capacity challenge the assumption that identical dietary substrates generate comparable bioactive metabolite exposure across individuals [42,43,44,45,46]. These uncertainties highlight the need to move beyond cross-sectional microbiome associations toward longitudinal and mechanistically informed human studies capable of linking microbial function, metabolite exposure, and standardized skin phenotypes. Accordingly, mechanistic plausibility and evidence of individual pathway components should be distinguished from direct demonstration of microbiome-mediated effects in humans.

Important translational challenges also remain regarding formulation stability, bioavailability, regulatory standardization, and clinical validation of microbiome-targeted nutricosmetic products [25,64,65,142,143,144,145,146,147,148]. Although probiotics, postbiotics, and microbiome-responsive interventions have shown biological effects in experimental and selected clinical settings, robust long-term clinical evidence supporting their efficacy in skin aging management remains limited [21,22,23,24,25,26,27,142,145,146]. Emerging technologies, including artificial intelligence (AI), multiomics integration, and microbiome engineering, offer exciting opportunities for personalized skin health management; however, ethical considerations, data interpretation challenges, and commercialization barriers require further attention [146,147,148,160,161].

**Figure 3:**
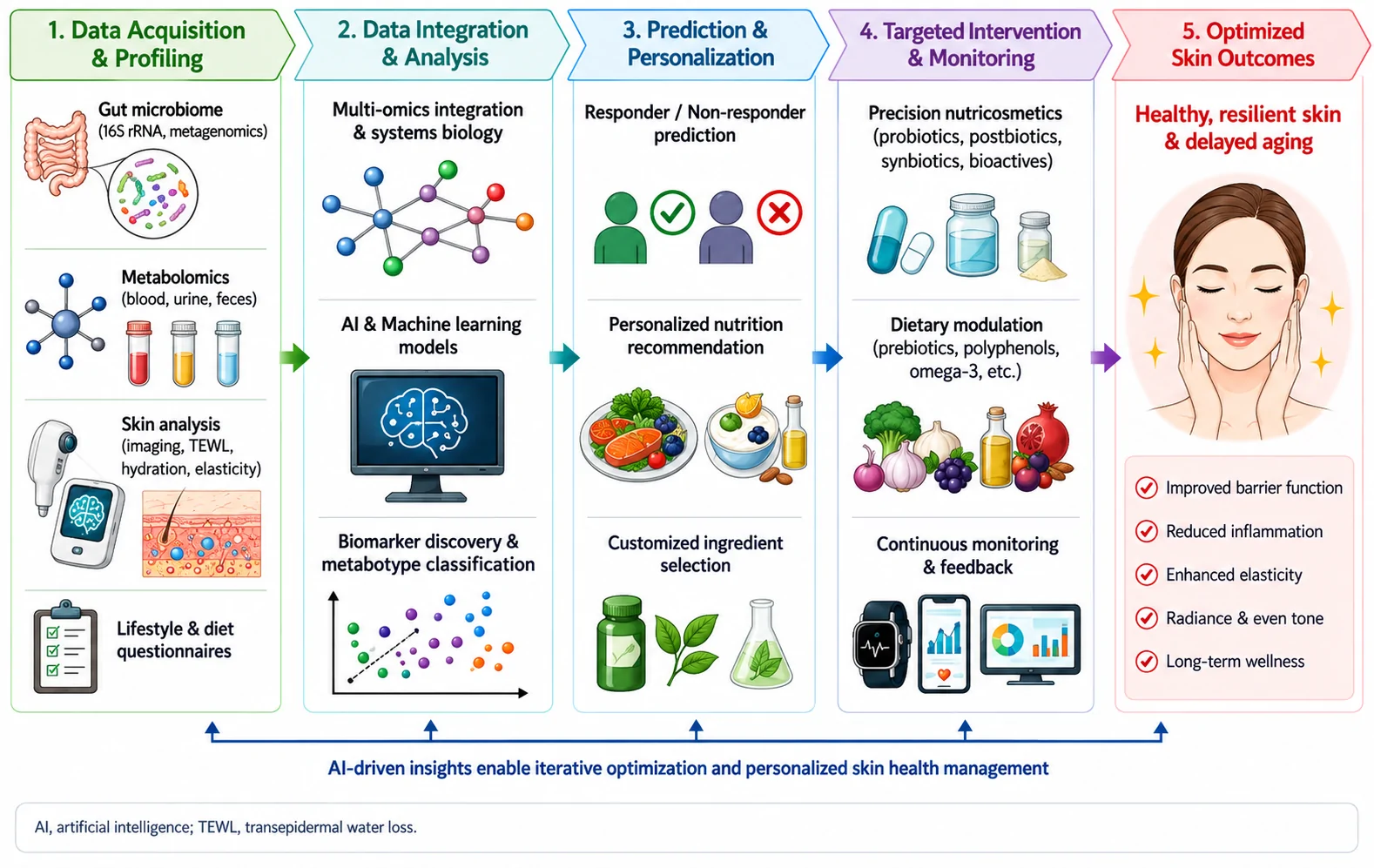
Emerging precision nutricosmetic and microbiome-responsive approaches for personalized skin health. Integration of microbiome profiling, microbial metabolite analysis, dietary information, multiomics technologies, and AI-assisted models may support the investigation of individualized microbiome-responsive strategies. These approaches remain emerging and require further clinical validation for application to skin health and healthy aging.

On the basis of these limitations, four research priorities are particularly important: (i) longitudinal and adequately powered human intervention studies integrating gut microbiome profiling with circulating microbial metabolite measurements and standardized skin-specific endpoints; (ii) mechanistic validation of candidate microbial metabolites using physiologically relevant concentrations and models capable of distinguishing direct from indirect skin effects; (iii) prospective stratification of participants according to baseline microbiome characteristics and metabotypes to identify reproducible responder and nonresponder phenotypes; and (iv) standardized multiomics and analytical frameworks that enable cross-study comparison and independent validation [15,16,17,18,19,115,145,146,147,148,149,150,151,152,153]. Addressing these priorities will be necessary to move the field from associative microbiome signatures toward mechanistically supported and clinically testable gut–skin relationships. Emerging precision microbiome-responsive strategies and future directions for personalized skin health management are summarized in Figure 3.

## 9. Conclusions

Current evidence supports biological links among dietary bioactives, gut microbial metabolism, microbiome-derived metabolites, and physiological processes relevant to skin health and aging. Mechanistic and preclinical studies have indicated that SCFAs, indole derivatives, phenolic metabolites, urolithins, bile acid derivatives, and lipid-associated metabolites can interact with pathways involved in inflammatory regulation, oxidative balance, epithelial homeostasis, mitochondrial function, and extracellular matrix remodeling [14,28,35,40,54,57]. Human intervention studies have also demonstrated that selected probiotics and oral nutricosmetic interventions can improve measurable skin outcomes [20,21,22,23,101,137,138]. However, these different levels of evidence should not be interpreted as equivalent support for a microbiome-mediated causal pathway.

Therefore, the principal conclusion of this review is not that microbiome-mediated effects of dietary bioactives on skin health are clinically established but that a substantial gap remains between mechanistic plausibility and demonstrated human causality. Evidence for individual components of the proposed diet–microbiome–metabolite–skin pathway is considerably stronger than evidence for the complete causal sequence. In particular, few human studies simultaneously assess dietary exposure or intervention, microbial alteration or biotransformation, relevant circulating microbial metabolites, and standardized skin-specific outcomes [16,17,18,19,20,21,22,23,24,25,26,27]. Consequently, clinical improvement following oral intervention should not be attributed to gut microbiome mediation solely on the basis of separate mechanistic, microbiome-association, or skin-efficacy studies.

This distinction is further complicated by substantial heterogeneity in study populations, intervention types, microbial strains, doses, treatment durations, analytical platforms, and skin-related endpoints [16,17,18,19,25,145,146,147,148]. Interindividual differences in microbial metabolic capacity and metabotypes introduce an additional source of variability because identical dietary substrates may not generate comparable metabolite exposure across individuals [42,43,44,45,46]. Moreover, context- and concentration-dependent effects of microbial metabolites and differences among *in vitro*, animal, observational, and interventional study designs limit direct extrapolation across evidence levels [14,28,35,38,54,55,56,57,58].

Future research should therefore focus on testing microbiome mediation rather than inferring it. Longitudinal and adequately powered human intervention studies should integrate dietary exposure, gut microbiome profiling, targeted measurement of microbiome-derived metabolites, mechanistic biomarkers, and standardized dermatological or biophysical outcomes within the same study design [15,16,17,18,19,115,145,146,147,148,149,150,151,152,153]. Prospective stratification according to baseline microbiome characteristics and metabotypes may further help distinguish microbiome-dependent responses from nonspecific interindividual variability. Until such evidence becomes available, many proposed diet–microbiome–metabolite–skin relationships should be regarded as biologically plausible and experimentally supported to varying degrees rather than as clinically established causal mechanisms. This evidence-centered distinction provides a more rigorous basis for evaluating microbiome-responsive nutricosmetic strategies and for defining the experimental requirements needed to advance the field from association toward causality.

## Figures and Tables

**Table 1:** Microbiome-derived metabolites involved in gut–skin communication and their mechanistic relevance to skin aging and nutricosmetics.

Metabolite Class	Representative Metabolites	Major Dietary/Microbial Origin	Major Targets or Pathways	Relevance to Skin Health and Aging	Evidence Level and Interpretation	Reference
Short-chain fatty acids	Acetate, propionate, butyrate	Fermentation of dietary fibers, resistant starch, mushroom polysaccharides	GPR41/43/109A, HDAC inhibition, AMPK, Nrf2, NF-κB	Barrier resilience, inflammatory balance, oxidative stress regulation, epidermal homeostasis	Preclinical mechanistic evidence; emerging human relevance	[28,29,30,31,32,33,34]
Butyrate-focused signaling	Butyrate	Butyrate-producing bacteria such as *Faecalibacterium*, *Roseburia*	Treg induction, HDAC inhibition, epithelial tight-junction regulation	Intestinal barrier support and indirect reduction in systemic inflammatory tone affecting skin	Preclinical mechanistic evidence	[31,32,33,34]
Tryptophan-derived indoles	Indole, indole-3-acetic acid, indole-3-propionic acid, indole-3-aldehyde	Microbial tryptophan metabolism	AhR, NF-κB, epithelial stress-response pathways	Keratinocyte differentiation, epithelial barrier regulation, immune balance, antimicrobial peptide expression	Preclinical mechanistic evidence; emerging human relevance	[35,36,37,38]
Kynurenine-related tryptophan axis	Kynurenine, kynurenic acid	Host–microbiome tryptophan metabolism	AhR, immune-metabolic regulation	May influence inflammatory skin disorders through immune regulation and barrier-associated pathways	Emerging/indirect evidence	[36,37,39]
Urolithins	Urolithin A, urolithin B	Microbial metabolism of ellagitannins and ellagic acid from pomegranate, berries, walnuts	Mitophagy, AMPK, Nrf2, mitochondrial quality control	Mitochondrial resilience, anti-aging biology, oxidative stress-associated skin protection	Preclinical mechanistic evidence; limited skin-specific human evidence	[40,41,42,43,44,45,46]
Simple phenolic metabolites	Protocatechuic acid, gallic acid, caffeic acid, ferulic acid, hydroxyphenylpropionic acids	Microbial degradation of flavonoids, anthocyanins, phenolic acids	Nrf2, NF-κB, MAPK-associated pathways	Antioxidant-associated skin support, inflammatory balance, ECM-related regulation	Preclinical mechanistic evidence	[42,47,48,49]
Isoflavone-derived metabolites	Equol	Microbial conversion of daidzein from soy isoflavones	Estrogen receptor-β, antioxidant pathways, collagen/elastin-related regulation	Skin aging relevance through estrogenic, antioxidant, and dermal structural support mechanisms	Human associative and preclinical mechanistic evidence; substantial interindividual producer variability	[50,51]
Lignan-derived enterolignans	Enterodiol, enterolactone	Microbial metabolism of plant lignans from flaxseed, whole grains, seeds	Phytoestrogenic signaling, antioxidant and immune regulation	Potential relevance to skin aging through hormone-related and antioxidant-associated pathways	Emerging/indirect evidence	[52,53]
Secondary bile acids	Deoxycholic acid, lithocholic acid, ursodeoxycholic acid derivatives	Microbial bile acid deconjugation and transformation	FXR, TGR5, VDR, inflammatory-metabolic signaling	Epithelial homeostasis, immune regulation, metabolic inflammation relevant to gut–skin communication	Emerging/indirect evidence	[54,55,56]
Lipid-derived microbial metabolites	Conjugated linoleic acid derivatives, hydroxy fatty acids	Microbial transformation of dietary fatty acids	PPARs, lipid signaling, inflammatory pathways	Epidermal lipid balance, inflammatory resolution, barrier-associated support	Emerging/indirect evidence	[57,58]
Omega-3-associated lipid mediators	EPA/DHA-derived lipid mediators, specialized pro-resolving mediators	Dietary marine lipids with host–microbiome interaction	Resolution pathways, NF-κB-related inflammatory regulation	Inflammatory balance, skin resilience, barrier support	Human and preclinical evidence; indirect microbiome-specific relevance	[58,59,60,61]
Microbial sphingolipids	Bacteroides-derived sphingolipids, ceramide-like molecules	Gut microbial lipid metabolism	Immune education, epithelial and lipid signaling	Possible relevance to epidermal lipid homeostasis and immune tone	Emerging/indirect evidence	[62]
Postbiotic peptides and exopolysaccharides	Bioactive peptides, EPS, cell-wall fragments	Fermentation-derived microbial products	Pattern-recognition receptors, epithelial signaling, immune regulation	Barrier support, skin microbiome balance, cosmetic/postbiotic formulation relevance	Emerging clinical/translational evidence	[10,63,64,65]
Aromatic amino acid metabolites	Phenylacetic acid, phenylpropionic acid derivatives	Microbial metabolism of phenylalanine/tyrosine and polyphenols	Redox and inflammatory signaling	Potential modulation of oxidative and inflammatory pathways relevant to skin aging	Emerging/indirect evidence	[35,66]
Potentially detrimental metabolites	*p*-Cresol, phenol, ammonia	Dysbiotic protein fermentation	Barrier-disruptive and pro-inflammatory pathways	May impair keratinocyte differentiation, hydration, and barrier integrity under dysbiosis	Limited human/preclinical evidence; adverse skin relevance reported	[67]
Trimethylamine-related metabolites	TMA, TMAO	Microbial metabolism of choline, carnitine, phosphatidylcholine	Systemic inflammatory and metabolic pathways	Indirect relevance through systemic inflammation and metabolic stress rather than direct skin effects	Emerging/indirect evidence; skin-specific evidence limited	[68]

Evidence assessment: Evidence was qualitatively categorized according to study design, directness to skin-related outcomes, consistency of findings, and translational relevance. Clinical evidence indicates support from human interventional studies directly evaluating relevant skin outcomes. Human associative evidence indicates evidence from observational or other human studies demonstrating associations without establishing causality. Preclinical mechanistic evidence refers primarily to animal and/or *in vitro* studies supporting biological mechanisms but with limited direct clinical validation. Emerging/indirect evidence indicates preliminary, limited, inconsistent, or indirectly extrapolated findings for skin physiology. Evidence consistency was qualitatively assessed on the basis of the agreement of findings across the cited studies, whereas major translational limitations included the availability of direct human validation, skin-specific outcomes, interindividual microbiome variability, and the degree of indirect extrapolation from experimental models. These categories are intended to provide a transparent narrative appraisal of the literature rather than a formal systematic evidence-grading system.

**Table 2:** Representative human intervention studies relevant to gut–skin communication and oral nutricosmetic strategies.

Intervention	Population and Study Design	Dose	Duration	Major Clinical/Biophysical Endpoints	Main Findings	Relevance to Gut–Skin Mechanisms	Ref.
*Lactobacillus plantarum* HY7714	110 adults aged 41–59 years with dry skin and wrinkles; randomized, double-blind, placebo-controlled trial	1 × 10^10^ CFU/day	12 weeks	Skin hydration, TEWL, wrinkle depth, skin gloss, elasticity	Improved skin hydration, wrinkle depth, gloss, and elasticity; greater suppression of TEWL than placebo	Direct microbiome-targeted clinical evidence; microbiome composition and microbial metabolites were not concurrently assessed	[20]
*Lactobacillus rhamnosus* SP1	20 adults with acne; randomized, double-blind, placebo-controlled pilot trial	3 × 10^9^ CFU/day (75 mg/day)	12 weeks	Clinical acne improvement; cutaneous IGF1 and FOXO1 expression	Improved acne appearance and altered insulin-signaling-related gene expression	Direct probiotic intervention; gut microbiome and microbial metabolites were not directly quantified	[21]
*Bifidobacterium lactis* CECT 8145 + *B. longum* CECT 7347 + *Lactobacillus casei* CECT 9104	50 children aged 4–17 years with moderate atopic dermatitis; randomized, double-blind, placebo-controlled trial	Total 1 × 10^9^ CFU/day	12 weeks	SCORAD index; topical corticosteroid use	Greater reduction in SCORAD and lower topical corticosteroid use than placebo	Direct microbiome-targeted intervention supporting clinical relevance in inflammatory skin disease; evidence remains strain- and population-specific	[22]
*Lactobacillus plantarum* PBS067 + *L. reuteri* PBS072 + *L. rhamnosus* LRH020	80 adults with mild-to-severe atopic dermatitis; randomized, double-blind, placebo-controlled trial	1 × 10^9^ CFU of each strain/day (3 × 10^9^ CFU total/day)	56 days	SCORAD, skin moisturization, smoothness, inflammatory parameters, subjective symptoms	Improved SCORAD, skin moisturization, smoothness, and inflammatory parameters	Direct probiotic clinical evidence; causal mediation through specific gut-derived metabolites remains unconfirmed	[23]
Specific collagen peptides	69 women aged 35–55 years; randomized, double-blind, placebo-controlled trial	2.5 or 5.0 g/day	8 weeks	Skin elasticity, moisture, TEWL, skin roughness	Significantly improved skin elasticity; effects on moisture and TEWL were less consistent	Systemic nutricosmetic evidence; microbiome mediation was not directly assessed	[101]
Low-molecular-weight collagen peptides	64 women aged 40–60 years with photoaged skin; randomized, double-blind, placebo-controlled trial	1000 mg/day	12 weeks	Skin hydration, elasticity, wrinkling	Improved skin hydration, elasticity, and wrinkling compared with placebo	Systemic oral efficacy; no direct demonstration of microbiome-mediated action	[137]
Astaxanthin + collagen hydrolysate	44 healthy adults with moderately photoaged skin; placebo-controlled clinical trial	Astaxanthin 2 mg/day + collagen hydrolysate 3 g/day	12 weeks	Facial elasticity, hydration, TEWL, procollagen I, MMP-1/-12, UV-induced DNA damage	Improved facial elasticity and TEWL; increased procollagen I and decreased MMP-1/-12 expression	Supportive systemic nutricosmetic evidence; gut microbiome involvement was not directly evaluated	[138]

Abbreviations: CFU, colony-forming units; FOXO1, forkhead box O1; IGF1, insulin-like growth factor 1; MMP, matrix metalloproteinase; SCORAD, scoring atopic dermatitis; TEWL, transepidermal water loss; UV, ultraviolet.

**Table 3:** Evidence-chain appraisal of representative diet–microbiome–metabolite–skin relationships.

Proposed Relationship	Mechanistic/Preclinical Evidence	Human Evidence	Concurrent Assessment of Microbiome/Metabolites and Skin Outcomes	Direct Human Microbiome-Mediated Causality	Critical Interpretation	Ref.
Dietary fiber/polysaccharides → SCFAs → immune/barrier regulation → skin outcomes	SCFA production from fermentable substrates and SCFA-mediated epithelial and immune regulation are well supported experimentally	Human relevance of SCFAs is established in broader physiological contexts, but skin-specific evidence is limited	Limited in the skin-related evidence reviewed here	Not established	Biological plausibility is supported, but direct demonstration of the complete dietary substrate → microbial SCFA production → systemic exposure → skin outcome pathway remains limited	[28,29,30,31,32,33,34]
Dietary tryptophan → microbial indoles → AhR signaling → epidermal/immune homeostasis	Strong mechanistic support for microbial tryptophan metabolism and indole–AhR signaling in epithelial and immune regulation	Skin-specific human evidence remains limited	Limited in the evidence reviewed here	Not established	Evidence is predominantly mechanistic and preclinical; translation of microbial indole signaling to clinically demonstrated human skin outcomes remains insufficient	[35,36,37,38]
Ellagitannins/polyphenols → urolithins → mitochondrial regulation → skin aging-related outcomes	Microbial urolithin formation and urolithin A-associated mitochondrial/mitophagy mechanisms are well characterized	Human studies demonstrate urolithin metabotypes and systemic biological activity, but direct skin-specific clinical evidence is limited	Not demonstrated for skin-specific outcomes in the representative evidence reviewed here	Not established	Evidence supports microbial biotransformation and systemic biological activity more strongly than a causal effect on human skin aging	[40,41,42,43,44,45,46,124]
Oral probiotics → gut microbial modulation → microbial/metabolic signaling → skin outcomes	Mechanistic and preclinical evidence supports immune-, barrier-, and microbiome-associated effects	Selected RCTs report improvements in hydration, TEWL, acne, and atopic dermatitis-related outcomes	Gut microbiome composition and/or microbial metabolites were not consistently assessed together with skin endpoints in the representative trials	Complete mediated pathway not established	Selected probiotics have human skin efficacy, but the extent to which these effects are mediated by gut microbial changes or specific metabolites remains unresolved and may be strain- and population-specific	[20,21,22,23]
Oral collagen peptides → proposed gut/microbiome interactions → skin outcomes	Peptide digestion, absorption, and potential host–microbiome interactions provide indirect mechanistic context	RCTs demonstrate improvements in selected skin hydration, elasticity, and wrinkle-related outcomes	Microbiome mediation was not directly evaluated in the representative skin trials	Not established	Clinical skin efficacy should not be interpreted as evidence of a microbiome-mediated mechanism	[101,102,103,104,137]
Astaxanthin/carotenoids → possible microbiome interaction → skin outcomes	Carotenoids have established skin-related biological effects, while interactions with gut microbial ecology and bioavailability have been proposed	Human intervention evidence supports selected skin outcomes for astaxanthin-containing interventions	Gut microbiome/metabolite mediation was not directly evaluated in the representative skin trial	Not established	Human skin benefits are relevant to oral nutricosmetics, but attribution to gut microbiome mediation remains unsupported by direct evidence	[92,93,94,95,96,138]

Evidence-chain appraisal: The table evaluates whether representative evidence supports sequential components of a proposed diet–microbiome–metabolite–skin pathway. “Direct human microbiome-mediated causality not established” indicates that the evidence reviewed here does not demonstrate, within the same human experimental framework, a sequential relationship linking dietary exposure or intervention, microbial alteration or biotransformation, relevant microbial metabolite exposure, and a skin-specific clinical or biophysical outcome. This appraisal is intended as a structured narrative assessment rather than a formal systematic evidence-grading or risk-of-bias analysis.

## Data Availability

No new data were created or analyzed in this study. Data sharing is not applicable to this article.

## Abbreviations

The following abbreviations are used in this manuscript:

AhR	Aryl Hydrocarbon Receptor
AI	Artificial Intelligence
AMPK	AMP-Activated Protein Kinase
ECM	Extracellular Matrix
EPS	Exopolysaccharides
FXR	Farnesoid X Receptor
GPR	G Protein-Coupled Receptor
HDAC	Histone Deacetylase
IL	Interleukin
LPS	Lipopolysaccharide
MAPK	Mitogen-Activated Protein Kinase
MMP	Matrix Metalloproteinase
mTOR	Mechanistic Target of Rapamycin
NF-κB	Nuclear Factor Kappa B
Nrf2	Nuclear Factor Erythroid 2-Related Factor 2
Parkin	Parkin RBR E3 Ubiquitin Protein Ligase
PGC-1α	Peroxisome Proliferator-Activated Receptor Gamma Coactivator-1 Alpha
PINK1	PTEN-Induced Putative Kinase 1
PPAR	Peroxisome Proliferator-Activated Receptor
PUFA	Polyunsaturated Fatty Acid
ROS	Reactive Oxygen Species
SASP	Senescence-Associated Secretory Phenotype
SCFA	Short-Chain Fatty Acid
SIRT1	Sirtuin 1
TGF-β	Transforming Growth Factor Beta
TGR5	Takeda G Protein-Coupled Receptor 5
Th17	T Helper 17 Cell
TMA	Trimethylamine
TMAO	Trimethylamine *N*-Oxide
TNF-α	Tumor Necrosis Factor Alpha
Treg	Regulatory T-Cell
TEWL	Transepidermal Water Loss
UV	Ultraviolet Radiation
VDR	Vitamin D Receptor
